# HaploBlocks: Efficient Detection of Positive Selection in Large Population Genomic Datasets

**DOI:** 10.1093/molbev/msad027

**Published:** 2023-02-15

**Authors:** Benedikt Kirsch-Gerweck, Leonard Bohnenkämper, Michel T Henrichs, Jarno N Alanko, Hideo Bannai, Bastien Cazaux, Pierre Peterlongo, Joachim Burger, Jens Stoye, Yoan Diekmann

**Affiliations:** Palaeogenetics Group, Institute of Organismic and Molecular Evolution (iomE), Johannes Gutenberg University, 55128 Mainz, Germany; Faculty of Technology and Center for Biotechnology (CeBiTec), Bielefeld University, Universitätsstr. 25, 33615 Bielefeld, Germany; Faculty of Technology and Center for Biotechnology (CeBiTec), Bielefeld University, Universitätsstr. 25, 33615 Bielefeld, Germany; Department of Computer Science, University of Helsinki, P.O 68, Pietari Kalmin katu 5, 00014 Helsinki, Finland; M&D Data Science Center, Tokyo Medical and Dental University (TMDU), 2-3-10 Kanda-Surugadai, Chiyoda-ku, Tokyo 101-0062, Japan; CNRS, Centrale Lille, UMR 9189, Univ. Lille, CRIStAL, F-59000 Lille, France; GenScale, Inria/Irisa Campus de Beaulieu, 35042 Rennes Cedex, France; Palaeogenetics Group, Institute of Organismic and Molecular Evolution (iomE), Johannes Gutenberg University, 55128 Mainz, Germany; Faculty of Technology and Center for Biotechnology (CeBiTec), Bielefeld University, Universitätsstr. 25, 33615 Bielefeld, Germany; Palaeogenetics Group, Institute of Organismic and Molecular Evolution (iomE), Johannes Gutenberg University, 55128 Mainz, Germany; Research Department of Genetics, Evolution and Environment, University College London, London WC1E 6BT, United Kingdom

**Keywords:** natural selection, genome scan, big data, population genetics

## Abstract

Genomic regions under positive selection harbor variation linked for example to adaptation. Most tools for detecting positively selected variants have computational resource requirements rendering them impractical on population genomic datasets with hundreds of thousands of individuals or more. We have developed and implemented an efficient haplotype-based approach able to scan large datasets and accurately detect positive selection. We achieve this by combining a pattern matching approach based on the positional Burrows–Wheeler transform with model-based inference which only requires the evaluation of closed-form expressions. We evaluate our approach with simulations, and find it to be both sensitive and specific. The computational resource requirements quantified using UK Biobank data indicate that our implementation is scalable to population genomic datasets with millions of individuals. Our approach may serve as an algorithmic blueprint for the era of “big data” genomics: a combinatorial core coupled with statistical inference in closed form.

## Introduction

Natural or Darwinian selection is one of the fundamental evolutionary processes shaping genetic variation, and the primary mechanism responsible for adaptation. At the molecular level, variants favored by natural selection due to the fitness advantage they confer are said to be positively selected. Positively selected variants are often relevant as they affected survival in the past.

In sets of contemporary genomes, positive selection is mainly inferred based on allele frequency differentiation, locally overrepresented ancestry, or genomic signatures expected under a selective sweep model. Numerous statistical approaches exist, descriptive (Alachiotis and Pavlidis 2018) and inferential (Stern et al. 2019) as well as mixed methods where summary statistics are computed on inferred structures such as identity-by-descent segments (Browning and Browning 2020) or genealogies (Speidel et al. 2019). Other frameworks rely on simulations under explicit demographic and/or sweep models, for example Approximate Bayesian Computation (Luqman et al. 2021) and Machine Learning that interprets detecting selection as a classification problem (Torada et al. 2019).

Development of new methods for detection of positive selection faces a challenge common to all branches of genomics: improving accuracy while keeping pace with the accelerating growth of genome databases. The sheer number of genomes is already a challenge for most methods, and future databases run the risk of being manageable only by very few approaches. At the moment, biobanks, for example, manage hundreds of thousands of human genomes (Bycroft et al. 2018), but the next generation of projects currently under way will see millions of individuals sequenced (Gaziano et al. 2016; All of Us Research Program Investigators et al. 2019).

Here, we extend a combinatorial pattern matching algorithm previously published by Alanko et al. (2020) and present HaploBlocks, a novel approach to swiftly scan large population genomic datasets for positively selected haplotypes. Our method performs model-based statistical inference, and estimates accurate selection coefficients *s*, here defined as the relative fitness advantage of a haplotype per generation, over the entire length of the mappable genome. We implemented HaploBlocks as open-source software in C++, freely available with basic usage documentation at https://github.com/bekirsch/HaploBlocks. Source code to replicate the validation and benchmarking presented below can be found at https://github.com/bekirsch/HaploBlocks-Evaluation.

## New Approaches

HaploBlocks works on phased chromosomes with no missing genotypes from multiple individuals, which are available for most genome databases including for example the UK Biobank (Bycroft et al. 2018). It proceeds in three main sequential phases (see Material and Methods for details).

After initial preprocessing, the first phase enumerates a simple combinatorial pattern we coin *maximal perfect haplotype block* (HB in short). A HB is defined as a set of rows and a start and end column, such that the substring defined by start and end column is the same in all specified rows. Additional maximality criteria are detailed in the Material and Methods section. Previously, Alanko et al. (2020) showed that HBs can be found in optimal linear time, and presented an algorithm based on the positional Burrows–Wheeler transform (pBWT) (Durbin 2014) that works efficiently also in practice.

By definition, HBs are identity-by-state segments shared across multiple chromosomes and assumed here to be identical-by-descent (IBD), that is inherited from a common ancestor. They are central for the second phase of HaploBlocks, as IBD segments are shortened via crossover events that accumulate at approximately constant rate per generation, and their length is therefore informative on the age of a block. We adapt a composite likelihood model presented in Chen et al. (2015) that integrates this “recombination clock” with a sweep model relating haplotype age and its observed frequency in the population to positive selection: the larger—and therefore younger—and more frequent a haplotype is, the stronger is the inferred selection. The simple structure of HBs allows for a closed-form derivative of the likelihood function, so that we estimate a maximum composite likelihood (MCL) selection coefficient $\hat{s}$ for every HB at nearly no additional computational cost.

The third phase implements two stringent filters that are applied sequentially and remove blocks that likely arose as a result of processes other than selection. Our sweep model assumes an infinite population, which amounts to ignoring genetic drift, and independence between haplotypes or equivalently a star topology, therefore resulting in a composite likelihood. We derive upper bounds for the age of HBs based on neutral population genetic models that account for common ancestry and genetic drift. First, we filter blocks comparing their age inferred by our model to expectations under the coalescent that are computed given haplotype length and absolute frequency. The impact on running time is kept minimal by use of a precomputed lookup table. Second, we compute the distribution of haplotype age given its relative frequency, using an approximation under the Wright–Fisher model presented in Slatkin and Rannala (2000). Blocks with age above a threshold are again discarded.

## Results

### Validation

We validate HaploBlocks by simulating a genomic region with a central allele under positive selection of varying strength with SLiM (Haller and Messer 2019) and msprime (Kelleher et al. 2016) (see Materials and Methods). In a first step, we evaluate how well presence and absence of selection is inferred, and secondly quantify how accurately selection coefficients are estimated.

**Fig. 1.**
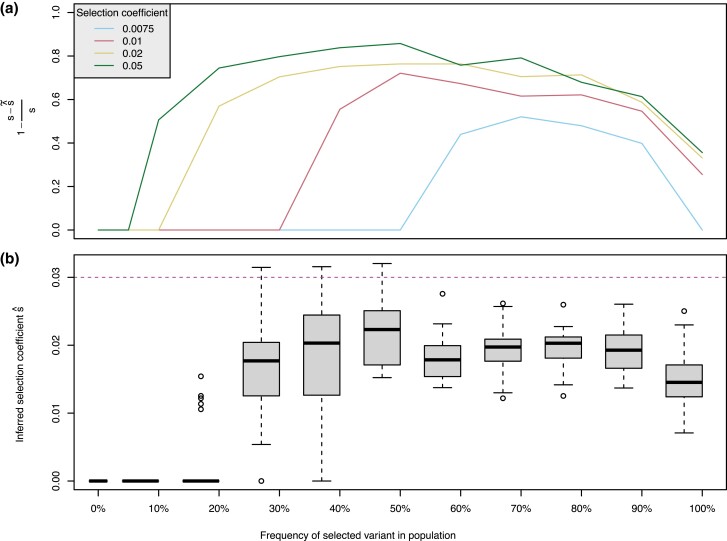
*Validation on simulated data.* (*a*) Median accuracy of 50 inferred selection coefficients $\hat{s}$ per simulated selection coefficient *s* ∈ {0.0075, 0.01, 0.02, 0.05} and per frequency in the population. (*b*) Inferred selection coefficients $\hat{s}$ for the European population from simulations of an Out-of-Africa model with a beneficial allele with selection coefficient *s* = 0.03.

Under a model of constant population size, we falsely infer selection in 1.5% (95% confidence interval (CI) [0.005, 0.04]) of the simulations (3 out of 200 simulations; supplementary figs. S5–S8, Supplementary Material online), where false positives are caused by HB with large but rare haplotypes that escape our filters and yield unrealistically high selection coefficient estimates (supplementary fig. S9, Supplementary Material online). Panel *a* in figure 1 summarizes the comparison of simulated with estimated selection coefficients for all blocks that pass our filters. We observe that the median estimated selection coefficient across 50 simulations at intermediate frequency of the selected allele in the population is roughly between 0.6 and 0.8 of the simulated value for coefficients between 1% and 5%. Three trends are apparent: first, the higher the simulated selection coefficient, the higher the estimation accuracy; second, the higher the simulated selection coefficient, the lower the minimum frequency of the selected allele in the population that allows for inference of a selection coefficient greater than zero; third, estimation accuracy drops with selected allele frequencies approaching fixation.

In addition, we test how well HaploBlocks performs under more complex demographic scenarios violating our assumption of a constant population size, by simulating data under bottleneck and migration models, and an Out-of-Africa model presented in Gravel et al. (2011), each with selection coefficients of 3%. For the bottleneck and migration model, we falsely infer selection in 10% (95% CI [0.03, 0.30]) and 20% (95% CI [0.08, 0.42]) of the simulations (2 and 4 out 20 simulations, respectively; supplementary figs. S12 and S14, Supplementary Material online). This is caused by HB that yield extremely high selection coefficient estimates (supplementary figs. S13 and S15, Supplementary Material online) also observed for the simulations with constant population size. No false positives are generated in the Out-of-Africa simulations (95% CI [0.0, 0.2]; 0 out of 16 simulations; supplementary fig. S11, Supplementary Material online). For the latter, neutral simulations are represented by 16 runs where the selected allele was lost over the course of the fixed number of generations specified by the demographic model (see supplementary fig. S10, Supplementary Material online for number of simulations per selected allele frequency bin). We find that the highest median accuracy of estimated selection coefficients is 0.6 of the simulated value achieved at intermediate allele frequency in the population, no selection is detected below a frequency of 10%, and the accuracy drops off as the alleles approach fixation (fig. 1*b*). Interestingly, while comparable overall, the accuracy for the simulated bottleneck and migration models at intermediate allele frequencies is better than for the constant model (supplementary figs. S13 and S15, Supplementary Material online).

### Benchmark

**Fig. 2.**
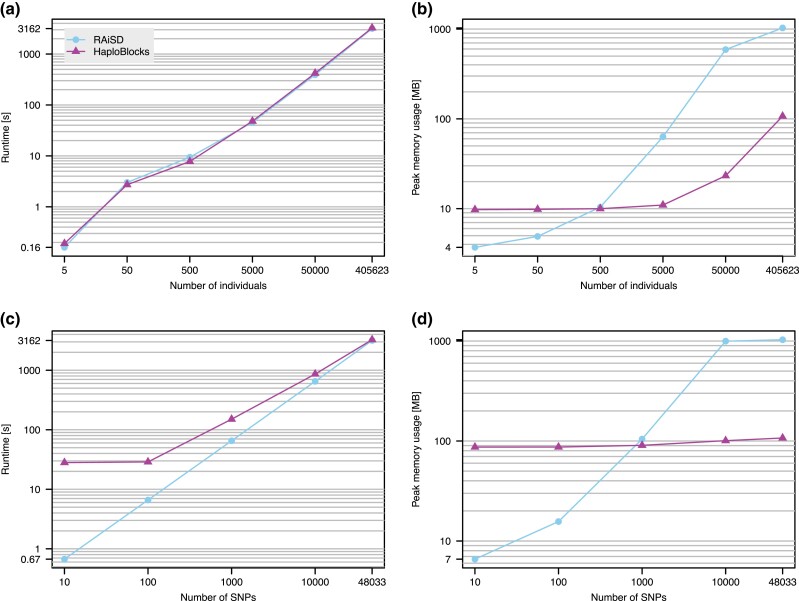
*Benchmark of HaploBlocks and RAiSD.* Runtime (*a*) and memory consumption (*b*) on 48,033 SNPs in an increasing number of individuals, and for an increasing number of SNPs (*c*,*d*) in 405,623 individuals.

Figure 2 (see also table 1) shows computational resource requirements measured on chromosome 2 of the UK Biobank genotype array data (Bycroft et al. 2018). Runtime increases linearly with the number of individuals and SNPs, but stays below one hour for the entire dataset consisting of 48,033 SNPs in 405,623 individuals. Memory consumption is only minimally influenced by the number of SNPs given a fixed number of individuals, here about 100 MB, and starts to exceed 10 MB on all SNPs only for datasets with more than 5,000 individuals.

**Table 1.** *Memory consumption and runtimes of RAiSD and HaploBlocks.* Table reporting peak memory consumption and runtimes of RAiSD and HaploBlocks for increasing number of individuals (rows 1 to 5) and increasing number of SNPs (rows 6–9). Values for the entire analysis dataset are given in the final row.

No. of individuals	No. of SNPs	Memory usage [MB]		Runtime [s]	
		RAiSD	HaploBlocks	RAiSD	HaploBlocks
5	48,033	3.704	9.748	0.16	0.19
50	48,033	4.904	9.820	3.01	2.72
500	48,033	10.396	9.984	9.41	7.76
5,000	48,033	63.112	10.964	45.56	48.09
50,000	48,033	590.840	23.252	391.49	420.47
405,623	10	6.584	86.964	0.67	28.08
405,623	100	15.652	87.0	6.6	28.83
405,623	1,000	104.544	90.516	65.28	150.3
405,623	10,000	996.332	100.78	650.7	862.58
405,623	48,033	1,029.888	107.124	3,161.99	3,255.3

We compare runtime and memory consumption of HaploBlocks against RAiSD (Alachiotis and Pavlidis 2018), which implements a model-free sliding-window approach computing a composite statistic sensitive to selective sweep signatures in the site frequency spectrum, levels of linkage disequilibrium, and genetic diversity estimates along chromosomes. We chose RAiSD as the authors benchmark computational efficiency and find it to be orders of magnitude faster than widely used alternative tools, while requiring only minimal memory. However, we stress that RAiSD does not perform inference and lacks a robust framework to evaluate significance of the resulting summary statistic, and HaploBlocks therefore generates at least conceptually superior output.

We achieve comparable performance with respect to runtime, although based on the slopes of the curves shown in panel *c* of figure 2 it appears that HaploBlocks may outperform RAiSD on datasets with more than 50k single nucleotide polymorphisms (SNPs). This is relevant as we analyzed genotype array data here, while whole-genome sequences have a much higher number of variable sites. HaploBlocks consumes less memory than RAiSD on datasets exceeding 500 individuals or 1000 SNPs, however, both tools use less than a GB and any difference is therefore negligible in practice.

### Genome Track

As our tool scans entire chromosomes, an intuitive and handy way to represent and interact with the results is via UCSC genome tracks (Raney et al. 2014). By default, HaploBlocks generates the necessary files to visualize estimated selection coefficients in their genomic context, serving as a starting point for example to pinpoint the actual allele favored by selection, its functional impact, and derive hypotheses about potential selective agents. This includes a filtering step that removes blocks ‘hidden’ behind others, which reduces file sizes and ensures that only the highest inferred selection coefficient is displayed at each position in the genome.

### UK Biobank Chromosome 2 Selection Scan

We scanned chromosome 2 of the genotype array data from the UK Biobank with HaploBlocks, and found 1,447,947 blocks after filtering and removing blocks overlapping large gaps in the assembly (see supplementary figs. S16–S19, Supplementary Material online for distributions of length, number of haplotypes in blocks and selection coefficients). Before removing blocks overlapping assembly gaps, a total of 2,332,653 out of 2,244,134,536 blocks pass both filters.

In order to assess how many blocks may be expected under neutrality, we simulated two datasets with the same number of SNPs as chromosome 2 of the UK Biobank genotype array (∼48 k) without introducing selection: first a sample from a large equilibrium population, and secondly from a demographic model with an ancient bottleneck and recent exponential population growth (see Materials and Methods for details on the simulations). In the equilibrium model, we found a total of 23,465,670 blocks, 88,906 (∼0.38%) of which pass both filters (see supplementary figs. S16, S17, and S20, Supplementary Material online for distributions of length and number of haplotypes in blocks). All blocks have estimated selection coefficients below 0.015, and most below 0.01 (supplementary figs. S18 and S22, Supplementary Material online). The model with bottleneck and exponential growth is more realistic, however, violates our assumption of a constant population size and we therefore expect a higher number of false positives as already observed in the validation. Indeed, we found a total of 18,327,632 blocks, 1,252,333 (∼6.83%) of which pass both filters (see supplementary figs. S16, S17, and S21, Supplementary Material online). Most blocks have estimated selection coefficients below 0.02, but few reach coefficients around 0.04 (supplementary figs. S18 and S23, Supplementary Material online). This suggests that a significant proportion of blocks that we find in the UK Biobank genotype array data are potential false positives. However, we repeated the analyses with approximately 17 times higher SNP density corresponding to full-genome sequencing data (see Materials and Methods), resulting in only 1 (∼0.00%) and 46,168 (∼0.1%) out of 56,444,051 and 44,998,854 blocks passing the filters for the constant and nonequilibrium model respectively (see supplementary figs. S16 and S17, Supplementary Material online). Besides two extremely large and rare haplotypes, all HBs have selection coefficients below 0.015 (supplementary fig. S18, Supplementary Material online). We therefore expect HaploBlocks to perform significantly better on full-genome sequences, especially when focussing on HBs with selection coefficient estimated to be above 0.015.

We quantified how much of chromosome 2 is covered by selected HaploBlocks at different selection strength (supplementary fig. S24, Supplementary Material online). While the high proportions for blocks including those with selection coefficient below 4% are inflated by false positives (supplementary figs. S25, S26, Supplementary Material online), we find ∼5% of the genome to be covered by strongly selected blocks not found in any simulation.

**Fig. 3.**
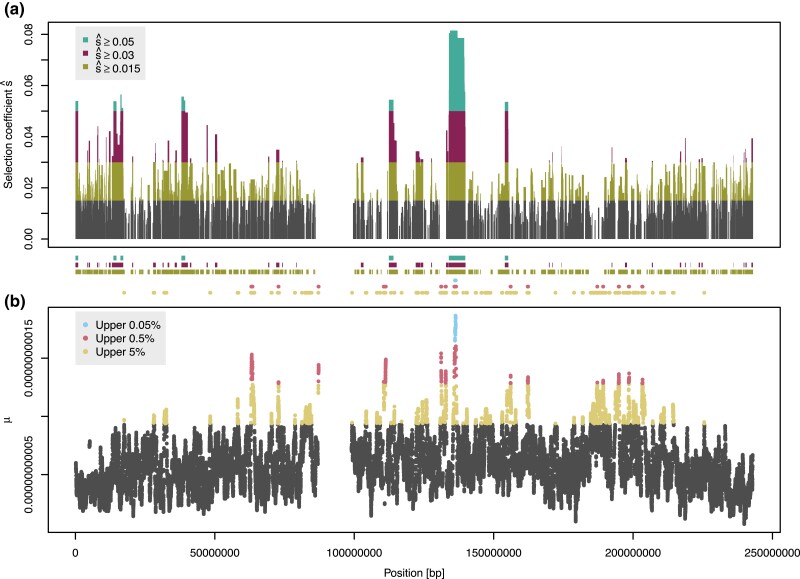
*Scan of UK Biobank chromosome 2.* Panel (a) shows the results of HaploBlocks; rectangles span the corresponding chromosomal region, with height displaying estimated selection coefficient. Regions with inferred selection coefficients above 5%, 3%, and 1.5% are highlighted. As shown in the main text, HB with selection coefficient below 1.5% are prone to be false positives due to the low SNP density of the genotype array. Panel (b) plots the μ-statistic computed by RAiSD with default parameters; regions corresponding to the top 0.05%, 0.5%, and 5% are highlighted. For better comparability, colored regions are also shown in-between panels. Telomeres and the centromere were masked for both HaploBlocks and RAiSD, and blocks intersecting with or overlapping large gaps in the hg19 assembly were removed. To maintain comparability, SNPs flanking telomeres and the centromere were removed from the RAiSD output.

Figure 3 summarizes the blocks found and compares the results to those obtained with RAiSD, however, we caution that Alachiotis and Pavlidis (2018) have not used or validated their tool on genotype array data. The highest selection coefficient is inferred for the locus harboring the European lactase persistence (LP) allele, consistent with it being among the strongest selected loci in humans (Ségurel and Bon 2017). Table 2 lists the genes overlapping the regions with selection coefficients inferred to be above 5%, and highlights those genes previously flagged by genome-wide selection scans. All regions found by HaploBlocks contain at least one such gene, with exception of a single chromosomal region that covers no genes at all. The strongest signal found by RAiSD is also located in the broader region around the LP locus (the European LP SNP rs4988235 itself is not part of the UK Biobank genotype array data), but overall there is no strong correlation between selection strength inferred by HaploBlocks and *μ*-statistic (supplementary fig. S27*a*, Supplementary Material online). Interestingly, RAiSD flags three loci with *μ*-statistics in the top 0.5% for which HaploBlocks infers no selection; while this is no formal demonstration, we note that these three loci lie in chromosomal regions with low recombination (supplementary fig. S27*b*, Supplementary Material online), a known confounding factor for RAiSD (Alachiotis and Pavlidis 2018).

**Table 2.** *Regions of chromosome 2 with inferred selection coefficient above 5% in the UK Biobank.* For the full profile of inferred selection coefficients, see figure 3. Coordinates are given in base pairs (hg19). Genes names are italicized, and bold if they have an entry in PopHumanScan (Murga-Moreno et al. 2019).

start	end	genes overlapping with the region
11,944	987,397	*SH3YL1, ACP1, ALKAL2, TMEM18, **SNTG2^+^**, FAM110C*
13,623,232	14,725,102	
16,127,607	17,140,887	FAM49A*
38,020,211	39,368,495	*RMDN2, CYP1B1, ATL2, **HNRNPLL^1^**, GALM, **SRSF7^1^**, GEMIN6, **DHX57^1^**, MORN2, ARHGEF33, **SOS1^1^***
112,421,035	114,063,686	*ANAPC1, MERTK, TMEM87B, FBLN7, **ZC3H8^a^**, **ZC3H6**, RGPD8, TTL, POLR1B, CHCHD5, SLC20A1, NT5DC4, CKAP2L, IL1A, IL1B, **IL37**, IL36G, IL36A, **IL36B**, IL36RN, IL1F10, IL1RN, PSD4, PAX8*
133,907,638	139,818,324	*NCKAP5, MGAT5, **TMEM163^a^**, **ACMSD**, **CCNT2**, **MAP3K19**, **RAB3GAP1**, **ZRANB3**, **R3HDM1**, **UBXN4**, **LCT**, **MCM6**, **DARS1**, CXCR4, THSD7B, HNMT, **SPOPL^a^**, NXPH2*
154,003,158	155,212,216	*RPRM, **GALNT13^2^***

+ gene is found in PopHumanScan, but is not listed if only the haplotype boundaries are specified.

* region overlapping the haplotype is present in PopHumanScan, but not the gene itself.

a no selection signature detected in Europeans populations, but in African or Asian.

1 found in African populations (Granka et al. 2021),

2 found in Southwestern Chinese (Liu et al. 2021).

The possibilities to validate the selection coefficients themselves are limited, crucially as approaches that estimate selection strength cannot handle hundreds of thousands of individuals. Though anecdotal, we note that—based on ancient DNA and a therefore independent analysis—selection strength on the European LP allele has recently been estimated to lie between 3% and 9% in Central/Northern Europeans (Burger et al. 2020). This brackets the 8% estimated by HaploBlocks.

## Discussion

We present HaploBlocks, an approach massively reducing the computational cost of model-based inference of selection coefficients in large population genomic datasets. We show our method is accurate (fig. 1), and scalable to datasets with millions of individuals (fig. 2). Currently, these database sizes are only manageable by very few approaches all based on summary statistics. We achieve this through efficient algorithmic design alone, without hardware acceleration techniques: HaploBlocks combines linear time combinatorial pattern matching based on the pBWT with statistical inference by closed-form MCL estimation, and uses approximations to efficiently filter blocks that likely arose due to processes other than selection.

On simulations, HaploBlocks infers very few false positives, that are all caused by HB with long but rare haplotypes yielding exceedingly high selection coefficient estimates. Both the power to infer presence of selection and the accuracy of estimates increase with selection strength and initially with haplotype frequency. We observe the tendency to slightly underestimate the simulated selection coefficients on average (fig. 1). In part, this is because our definition of HB does not capture the full extent of individual haplotypes, but only the overlap between all of them, which overestimates the age of a block. However, this approximation is crucial as it allows for a closed-form derivative of the likelihood function and therefore its efficient computation.

Another accuracy-speed tradeoff is introduced by the requirement of perfect matching, as opposed to approximate matching tolerating some mismatches in a HB, which is harder to solve and therefore slower (Williams and Mumey 2020). Perfect matching renders HBs vulnerable for example to sequencing errors and recent mutations, which both break up blocks leading to the underestimation of selection strength. To mitigate the problem, we recommend filtering by genotype quality and removal of low frequency alleles in the preprocessing step. This effect also explains the drop in prediction accuracy for high-frequency HBs observed in figure 1: HBs accumulate mutations with age, especially towards the end of the frequency trajectory as the speed of convergence towards fixation slows.

HaploBlocks performs statistical inference based on a selective sweep model, yet, we made numerous simplifying assumptions for the benefit of speed. For example, we focus on additive selection, as allele frequency trajectories under dominant and recessive models cannot be approximated by a sigmoidal curve; we do not model phenomena like background selection or variable selection strength; and we assume the simplest possible demography, a constant, infinite population size. An infinite population size amounts to ignoring the effects of genetic drift, which becomes reasonable the stronger selection is. Note that we indirectly account for drift via our second filter, which rejects HBs old enough to have risen to their current frequency by drift alone. The assumption of a constant population size is rarely realistic, and nonequilibrium demography has previously been identified as an important confounding factor (Jensen et al. 2005). While we do not systematically explore the effects of violations of our demographic model, we simulated simple bottleneck and migration models and a more complex Out-of-Africa demography. Despite elevated false positive rates in the former (supplementary figs. S12 and S14, Supplementary Material online), HaploBlocks is robust and the accuracy of selection coefficient estimates is not systematically affected (figs. 1*b*, supplementary figs. S13 and S15, Supplementary Material online).

The strength of our approach is its scalability to large datasets, and we therefore showcase HaploBlocks by applying it to one of the currently largest human genome databases, the UK Biobank. However, we note that as long as genomes are phased and sampled from a contemporaneous population, either ancient or extant, our tool also works on genomes from other species, including those with different ploidies. Here we scan chromosome 2 of the UK Biobank for positive selection and compare the results to those produced by RAiSD. We note that both the inferred proportions of the chromosome covered by haplotypes under selection and the estimated selection coefficients are generally high, which is due to the relatively sparse sampling of variants in the UK Biobank array data leading to longer haplotypes. We expect overall fewer HBs with lower selection coefficients on whole-genome sequencing data with many more variants. The advantages of HaploBlocks’ output over summary statistics are at least threefold: as we estimate an interpretable parameter, the selection coefficient, there is no need to restrict the analysis to top hits, therefore generating a result at every position in the genome; as HBs can comprise only a fraction of haplotypes in the population sample, we potentially also find more recent selection that does not yet affect statistics computed over the entire dataset; moreover, as haplotypes are flagged and not only SNPs, important phenomena like hitchhiking of disease-causing variants important for evolutionary medicine can be studied directly. The fact that the top hits of our analyses were already identified in previous studies (table 2) demonstrates the plausibility of our results.

Besides presenting a scalable tool for detecting positive selection, our layered algorithmic design—fast MCL estimation on top of efficient combinatorial pattern matching—may serve as a promising paradigm for the “big data” era of genomics.

## Materials and Methods

### Preprocessing

HaploBlocks uses the uncompressed VCF file format (Danecek et al. 2011), with arbitrary polarization and masked cetromeres and telomeres. The VCF file has to be phased, imputed or contain no missing genotypes, and filtered for biallelic SNPs only. In the case of whole-genome sequencing data, we additionally recommend to filter out SNPs with minor allele frequency below 1%, as they tend to break up HBs despite them being IBD. The genotypes are then converted to a binary matrix.

Besides the VCF file, a genetic map is required as input, provided in PLINK map format (Chang et al. 2015), that is one line per variant containing the chromosome code, variant ID, and positions in base pairs and centimorgans.

HaploBlocks expects that the effective population size is specified, which affects the frequency chosen to correspond to a single haplotype (see Eqs. 6, 9, and 17), and therefore the estimated selection coefficients and stringency of the filters. We exemplify the effect in figure S28, showing that lower effective population size leads to lower estimates of the selection coefficient and stricter filtering. In the case of uncertainty about the effective population size of a population under study, we therefore recommend choosing values at the lower end of the probable range as a conservative choice. While HaploBlocks works under the simplifying assumption of an equilibrium demography, the effective population size parameter offers the possibility, albeit limited, to introduce some prior knowledge about demography, in case of bottlenecks or expanding populations for example via the geometric mean of effective population sizes over time. Unless specified differently, we use an effective population size of 10,000 diploid individuals throughout the paper.

In a separate step before running the main algorithm, HaploBlocks generates a lookup table precomputing the first percentile of the distribution of time to the most recent common ancestor for a specified effective population size and varying number and lengths of haplotypes in a HB. These values are used for the first filter based on recent common ancestry. The table needs to be computed only once per effective population size and can be reused across runs, and reduces computation time for the quantile to a simple lookup or linear interpolation in case of intermediate values not present in the table.

### Enumerating Haplotype Blocks

**Fig. 4.**
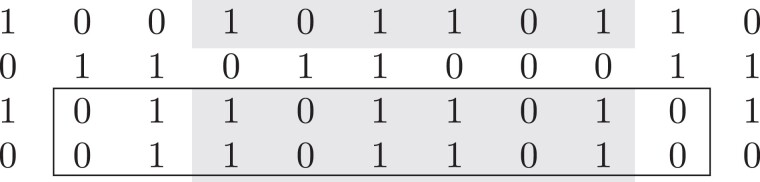
Illustration of the definition of maximal perfect haplotype blocks. Shown are four sequences of length 11. One block, shaded in gray, covers six variable sites in three of the four sequences, another one, indicated by the black box, covers nine sites in two sequences. Both haplotype blocks are maximal, that is, they cannot be extended to the left, to the right, or by an additional row.

A maximal perfect haplotype block (HB) for *k* haplotype sequences *S* = (*s*_1_, …, *s*_*k*_) of the same length *n* is a triple (*K*, *i*, *j*) where *K* is a subset of the given haplotype sequences, *K*⊆{1, …, *k*}, |*K*| ≥ 2, and 1 ≤ *i* ≤ *j* ≤ *n* such that for each sequence *s* ∈ *K* the interval *s*[*i*, *j*] is identical (*equality*) and the HB cannot be extended to the left (*left-maximality*), to the right (*right-maximality*) or by an additional haplotype (*row-maximality*) without violating the equality property (Alanko et al. 2020). An example of two HBs in a set of four haplotype sequences is given in figure 4. Note that the definition of HBs is not restricted to binary alleles, although our implementation currently supports only binary haplotype sequences.

Alanko et al. (2020) show that HBs can be identified in optimal, linear time by an algorithm that uses the positional Burrows–Wheeler Transform (Durbin 2014), a data structure that has proven useful in several applications in haplotype sequence analysis.

The algorithm constructs in linear time the two arrays *a*_*j*_ and *d*_*j*_ of the pBWT of *S* on the fly column by column for *j* = 1, …, *n*, where *a*_*j*_ is a permutation of {1, …, *k*} with $s_{a_{j}[1]}[1..j]\leq \cdots \leq s_{a_{j}[k]}[1..j]$ colexicographically (i.e., right-to-left lexicographically) and *d*_*j*_[*r*] is the starting point of the longest common suffix of $s_{a_{j}[r]}[1..j]$ and $s_{a_{j}[r-1]}[1..j]$ for 1 < *r* ≤ *k* and—by convention—*d*_*j*_[1] = *j* + 1. Then, when *a*_*j*_ and *d*_*j*_ are available, the set *B*_*j*_ of HBs that end at column *j* can be identified as quadruples (*i*, *j*; *x*, *y*) with 1 ≤ *i* ≤ *j* and 1 ≤ *x* < *y* ≤ *k* such that *d*_*j*_[*r*] ≤ *i* for all *r* ∈ {*x* + 1, …, *y*} *(equality)*, there exists at least one *r* ∈ {*x* + 1, …, *y*} such that *d*_*j*_[*r*] = *i (left-maximality)*, and *d*_*j*_[*x*] > *i* and *d*_*j*_[*y* + 1] > *i (row-maximality)*. In addition, *right-maximality* needs to be tested for each such HB candidate, which is done by building a bit vector *V*_*j*_ indicating changes in the next column of *S* and another vector of prefix sums of *V*_*j*_. Querying this sum vector allows to test right-maximality for any HB candidate in constant time. Since the pBWT with the two additional vectors can be created in *O*(*k*) time for each of the *n* columns and the number of HB candidates ending in any column of the pBWT is at most *k* (Alanko et al. 2020), the overall run time is optimal *O*(*nk* + *z*) in the worst case, where *nk* is the size of the input and *z* is the size of the output.

### Population Genetic Model and Inference Scheme

We assume that a set of *k* chromosomes sampled from a randomly mating population is sufficiently large such that haplotype frequency in the population may be approximated by the observed frequency *y* = |*K*|/*k*.

For each maximal haplotype block (*K*, *i*, *j*), physical positions corresponding to indices *i* and *j* are converted from base pairs to genetic distance *d* quantifying genetic linkage in centimorgan, which is the chromosomal distance for which the expected number of crossovers in a single generation is 0.01. Distance value *d* in turn is converted to the recombination fraction *r*—defined as the ratio of the number of recombined gametes between two chromosomal positions to the total number of gametes produced—using Haldane’s map function (Haldane 1919)

(1) $$r=\frac{1-\exp\;(-\frac{2d}{100})}{2}$$

To obtain a likelihood function L(s|r,y) that can be maximized, where *s* is the selection coefficient, we follow and extend the composite likelihood approach presented by Chen et al. (2015). The central building block given a HB is the probability of no recombination event happening that would break a haplotype up, which is approximated in Chen et al. (2015, eq. 14) by:

(2) $$C(s,r,y)=e^{-rt}{\left(1-y_{0}(1-e^{st})\right)}^{r/s}$$

with initial haplotype frequency *y*_0_ at time 0, and time *t* defined as

(3) $$t=\frac{1}{s}\ln\;\left(\frac{y(1-y_{0})}{y_{0}(1-y)}\right).$$

In practice, we set *y*_0_ = 1/(2*N*_*e*_) corresponding to a single haplotype. We define the probability of a single haplotype within a HB as the result of two independent recombination events happening on either side of a conserved middle stretch (supplementary fig. S1, Supplementary Material online). One recombination event has a probability given by equation (2) for no event in the conserved stretch times one minus equation (2) for one or more effective recombinations at the border. We assume the recombination fraction between two contiguous SNPs, Δ*r*, to be small and constant, and can therefore write the composite likelihood of *s* given *k* haplotypes in a HB assumed to be independent without iterating over multiple terms as:

(4) $$L(s|r,y)={\left(C(s,r,y)\cdot (1-C(s,\Delta r,y))\right)}^{2k}.$$

In practice, we set Δ*r* to the recombination fraction corresponding to the mean distance between consecutive SNPs. The effect of this approximation is negligible, as Δ*r* is usually very small. We note that because HBs do not consider the full varying extent of the individual haplotypes, the estimate maximizing equation (4) systematically but conservatively underestimates the strength of selection.

Based on the likelihood equation (4), we derive a closed-form expression for the MCL estimate $\hat{s}$ of the selection coefficient (see Supplementary Material online for details). By taking the logarithm and after some algebraic transformations, we get

(5) $$\begin{array}{rl} & \ln\;L(s\;|\;r,y)\\ & \;=2k\left(\frac{r}{s}\ln\;\left(\frac{y_{0}}{y}\right)+\ln\;\left(1-{\left(\frac{y_{0}}{y}\right)}^{\Delta r/s}\right)\right). \end{array}$$

In order to maximize, we take the derivative of equation (5) and determine the optimum by equating to zero, which yields

(6) $$\hat{s}=\frac{\Delta r}{\ln\;\left(\frac{r}{\Delta r+r}\right)}\cdot \ln\;\left(\frac{y_{0}}{y}\right).$$

Note that by substituting $\hat{s}$ into equation (3), we obtain an estimate $\hat{t}$.

### Filtering

We implement two filters that aim to remove HBs that may be explained by genetic drift or recent common ancestry rather than natural selection. See the Supplementary Material online for details on the choice of thresholds.

To account for genetic drift, we derive an upper bound on the age of HBs, which is based on the Wright–Fisher model and solely on haplotype frequency, that is does not consider haplotype length. Age is defined as the time since the last mutation event created the allelic sequence of a HB. The cumulative distribution function of allele age *t*_1_ for neutral alleles in equilibrium populations may be approximated by (Slatkin and Rannala 2000, eq. 6):

(7) $$Pr(t_{1}\leq t)=(1-p{)}^{-1+n/(1+nt/2)}$$

with *p* being the frequency of a haplotype in a sample of *n* chromosomes. We obtain the quantiles by equating the right side of equation (7) to *q*, yielding

(8) $$t_{1}(q)=\frac{2\ln\;(1-p)}{\ln\;(q)+\ln\;(1-p)}-\frac{2}{n}.$$

Next, we use the frequency *y*, the selection coefficient estimate $\hat{s}$ of a reported haplotype block to compute an estimate ${\hat{t}}_{1}$ for the haplotype age under selection from equation (3) with *y*_0_ = 1/(2*N*_*e*_):

(9) $${\hat{t}}_{1}=\frac{1}{\hat{s}}\ln\;\left(\frac{y\left(1-\frac{1}{2N_{e}}\right)}{\frac{1}{2N_{e}}\left(1-y\right)}\right)$$

If it is unlikely that the observed haplotype frequency is due to genetic drift given the young estimated age of a haplotype, we keep the block. We parametrize the quantile threshold by a minimum reportable selection coefficient *s*_min_

(10) $$q(s_{\min},N_{e},p)=\min\left(0.01,\max\left(0.0001,q^{\prime }\right)\right)$$

where

(11) $$q^{\prime }={\left(1-p\right)}^{-1+n/(1+nt^{\prime }/2)}$$

and

(12) $$t^{\prime }=\frac{1}{s_{\min}}\cdot \ln\;\left(\frac{\;p\cdot (1-\frac{1}{2\cdot Ne})}{\frac{1}{2\cdot Ne}\cdot (1-p))}\right).$$

Therefore, we apply an adaptive threshold between 0.01% and 1%, and selection is only inferred for blocks for which ${\hat{t}}_{1}<2N_{e}\cdot t_{1}(q(s_{\min},N_{e},p))$ holds. Older blocks are discarded from the output, as genetic drift cannot be dismissed. The preceding factor results from *t*_1_ being given in units of 2*N*_*e*_ generations.

A second threshold aims at removing blocks that are conserved due to recent common ancestry. We again derive a threshold for the age of HBs, based on the probability distribution of the time to the most recent common ancestor *t*_MRCA_. In the Kingman coalescent framework and under a hypothesized effective population size *N*_*e*_, the probability distribution of the *t*_MRCA_ of a given haplotype block (*r*, *y*, *k*) can be expressed as the sum and therefore convolution of independent exponential random variables (Donnelly et al. 1996; Pagani et al. 2018)

(13) $$\begin{array}{rl} & Pr(t_{\mathrm{MRCA}}\;|\;r,N_{e},k)\\ & \;=\sum_{i=2}^{k}\left(\lambda_{i}\;e^{-\lambda_{i}t}\prod_{\;j=2,j\neq i}^{k}\frac{\lambda_{j}}{\lambda_{j}-\lambda_{i}}\right) \end{array}$$

with *λ*_*i*_ = *i*(*i* − 1 + 2*N*_*e*_  *r*)/(2 *N*_*e*_), *i* ∈ {2, …, *k*}.

We use a single gamma distribution for approximating equation (13) following Covo and Elalouf (2014, Theorem 2.1) which provides additional computational efficiency and numerical stability. This is possible because an exponentially distributed random variable *X* ∼ Exp(*λ*) with rate parameter *λ* is equivalent to a gamma-distributed random variable *X* ∼ Gamma(1, *λ*^−1^) with shape parameter *α* = 1 and rate parameter *β* = *λ*^−1^.

Let $\beta_{i}=\lambda_{i}^{-1}$, with *i* ∈ 2, …, *k*, be the scale parameters, then parameter $\hat{\beta }$ of the single gamma approximation is the solution *β* > 0 of the equation

(14) $$\frac{\mu }{2}-2\sum_{i=2}^{k}\frac{\beta_{i}^{3}}{(\beta_{i}+\beta {)}^{2}}=0$$

with lower and upper bounds

(15) $$\frac{\mu }{k-1}\leq \hat{\beta }\leq \max_{i}(\beta_{i})$$

and with

(16) $$\mu =\sum_{i=2}^{k}\beta_{i}.$$

Scale and shape parameters are given by $\hat{\beta }$ and $\mu /\hat{\beta }$, respectively. For a comparison of the exact and approximated *t*_MRCA_, see figure S2.

As our model does not allow to estimate *t*_MRCA_ for a given HB, we approximate it by the time at which two alleles are present, denoted *t*_2_. Note that *t*_1_ > *t*_2_ ≥ *t*_MRCA_ holds, justifying the decision to prefer *t*_2_ over *t*_1_ as an approximation for *t*_MRCA_.

${\hat{t}}_{2}$ can again be obtained from equation (3) with *y*_0_ = 2 · 1/(2*N*_*e*_) = 1/*N*_*e*_. Substituting into equation (3) yields

(17) $${\hat{t}}_{2}=\frac{1}{\hat{s}}\ln\;\left(\frac{y\left(1-\frac{1}{N_{e}}\right)}{\frac{1}{N_{e}}\left(1-y\right)}\right).$$

We set the threshold to the first quantile of the gamma distribution and remove blocks with ${\hat{t}}_{2}$ estimates that are younger.

### Postprocessing

In order to avoid reporting false positives, we remove HBs from the output that intersect with or overlap large gaps in the hg19 assembly, as provided for example by the UCSC genome browser.

### Validation

We ran 200 simulations of a chromosomal region with an allele under positive selection added to the centre to evaluate our method. Each simulation was performed with 2,000 artificial chromosomes sampled at 13 frequencies, resulting in a total of 26,000 artificial chromosomes per simulation. We implemented a hybrid approach combining forward simulations in SLiM (Haller and Messer 2019) with coalescent simulations in msprime (Kelleher et al. 2016). This strategy allows to efficiently implement an initial neutral phase, or “burn-in,” in order to reach mutation-drift equilibrium before introducing non-neutral dynamics. Relying solely on forward simulations for the burn-in phase is time-consuming as the entire population—including individuals not ultimately part of the sample—has to be simulated.

Our simulation approach is similar to the one outlined in Haller et al. (2019, Example 4). Every run starts with a forward simulation of a Wright–Fisher population of 10,000 diploid individuals and a genomic region spanning 10 Mb in SLiM. A beneficial mutation with selection coefficient *s* ∈ {0.0075, 0.01, 0.02, 0.05} is introduced at the center of the artificial chromosome at 5 Mb. The recombination rate per site per generation is set to 1.0 × 10^−8^, and an output is generated at frequencies *y* ∈ {0, 0.02, 0.05, 0.1, 0.2, 0.3, 0.4, 0.5, 0.6, 0.7, 0.8, 0.9, 1}. After simulating the selective sweep in SLiM, a chromosome-wide genealogy is simulated for every individual with msprime. Finally, neutral mutations are randomly added to the branches of the tree-sequence under an infinite-sites model (Kimura 1969) with mutation rate per site 2.5 × 10^−8^ (Nachman and Crowell 2000). Finally, 1,000 diploid individuals are sampled for each selection coefficient *s* and frequency *y*.

Additionally, we ran 500 simulations with an initial 14,620 artificial chromosomes each, constituting the founding population in the out-of-Africa model of Gravel et al. (2011) (see estimated parameters **N*_*A*_* in table 2 therein). Again, we used the hybrid simulation approach described previously. A beneficial allele with selection coefficient *s* = 0.03 is introduced in the European population at several time points after the bottlenecks. As simulations were not conditional on final frequencies, a varying number of simulations ended up in the eleven frequency intervals ([0%],(0%−10%],(10%−20%],…,(90%−100%]) shown in figure S10. Lastly, we sample 1,000 diploid individuals from each of the European populations.

Analogous to the previous simulations, we simulated two more models with 20 independent runs each. First a bottleneck model, for which we reduced the initial population size of 20,000 artificial chromosomes during burn-in to 5% for 10 generations. We then simulated 1,120 generations, approximately corresponding to the time between the African–Eurasian and the subsequent Asian-European population split according to the Gravel model. Second, a migration model for which we again simulated a burn-in for 20,000 artificial chromosomes, before splitting the population into two equally sized subpopulations (again 10,000 diploid individuals per population). We set the migration rates between the two subpopulations to 3.11 × 10^−5^ following the migration rates between Asian and European populations in the Gravel model, and simulated 1120 generations. In both models, a beneficial allele with selection coefficient *s* = 0.03 is introduced, and 2,000 artificial chromosomes are sampled at frequencies *y* ∈ {0, 0.02, 0.05, 0.1, 0.2, 0.3, 0.4, 0.5, 0.6, 0.7, 0.8, 0.9, 1}.

Confidence intervals for the proportion of *a* > 0 false positives in *n* simulations are computed as the 2.5th and 97.5th quantile of the beta distribution Beta(1 + *a*, 1 + (*n* − *a*)), or the 95th quantile for *a* = 0.

### Benchmark and UK Biobank Selection Scan

The files used for benchmarking and the selection scan on chromsome 2 are provided by UK Biobank in BGEN format. These are converted to the VCF format with BGENIX, a tool included in the BGEN library (Band and Marchini 2018). We remove samples flagged as closely related by UK Biobank, as well as individuals who requested to be excluded from the dataset, leaving 405,623 out of 487,409 individuals. Input VCF files are filtered to keep only biallelic variants with minor allele frequency above 1%.

For the analysis of the UK Biobank, we use a population-specific genetic map for British in England and Scotland (GBR) published in Spence and Song (2019).

RAiSD was installed according to the documentation provided at https://github.com/alachins/raisd and run with default parameters on the same architecture and with the same input files as HaploBlocks. Larger files containing more individuals require an increase of the maximum memory allowance in the RAiSD source code.

In order to assess the expected distribution of false positives in our analysis of the UK Biobank data, we simulated a dataset under neutral evolution comparable to the UK Biobank. The current autosomal effective population size of the UK Biobank has most recently been estimated to be 10^7^ (Cai et al. 2022, figs. 3–5), and the 405,623 individuals individuals analyzed here therefore correspond to ∼4%. As the computational resources for a simulation of that size are excessive, we instead sampled 4,000 individuals from a simulation with a population size of 10^5^. We used the exact same approach as in the simulations for validation, with a chromosome length of 242,193,530 bp. We set the recombination rate to 7.7 × 10^9^ to closely match the ∼187 cM of chromosome 2 (Spence and Song 2019). We filtered for minor allele frequency above 1%, and sampled the resulting 11,146,258 SNPs down to 48,033 as for the UK Biobank genotype array data analyzed here in a way to match the allele frequency spectrum of the original. In addition, we generated a second version randomly sampling the SNPs down to 800,664, corresponding to the number of SNPs with minor allele frequency above 0.01 found in chromosome 2 of the UK10k data (UK10K Consortium et al. 2015), in order to emulate full sequencing data. We computed a lookup table for effective population size 10^5^ and set the parameter of HaploBlocks accordingly for the analysis of the simulation.

Furthermore, we simulated a second dataset under a neutral nonequilibrium model, inspired by Gravel et al. (2011) and intended to match the demography of the UK Biobank population more closely. Instead of a constant population size we started 5,921 generations ago with 28,948 artificial chromosomes and introduced a bottleneck 2,056 generations ago reducing the population size to only 3,722 artificial chromosomes. We introduced an exponential growth rate at 0.4247, resulting in a final population size of 10^5^ in the present generation. Again, 4,000 individuals were sampled for the analysis and additional steps were performed as described above, including downsampling the number of SNPs matching the allele frequency spectrum of the UK Biobank data. We also analyzed the full dataset consisting of 721,189 SNPs without downsampling. Under this demographic model the geometric mean of the effective population size is 9741, which we used for the lookup table and as HaploBlocks parameter for the analysis of this simulation.

## Supplementary Material

msad027_Supplementary_Data
