# Supplementary material for: HaploBlocks: Efficient Detection of Positive Selection in Large Population Genomic Datasets
Source: Mol Biol Evol. 2023 Feb 15;40(3):msad027. doi: 10.1093/molbev/msad027 (PMC9985328; doi:10.1093/molbev/msad027)
Supplement: msad027_Supplementary_Data [file msad027_supplementary_data.pdf]

SUPPLEMENTARY MATERIAL

Population genetic model and inference scheme—derivation of Equation 6

We start by expanding the logarithm of Equation 4, yielding

$$\begin{aligned}\ln\mathcal{L}(s|r,y) = 2k\Bigg(&\left(-rt+\frac{r}{s}\ln(1-y_0(1-e^{st}))\right)\\ &+\ln\left(1-e^{-\Delta rt}(1-y_0(1-e^{st}))^{\frac{\Delta r}{s}}\right)\Bigg),\end{aligned} \quad \text{(Supplementary Equation 1)}$$

with time

$$t=\frac{1}{s}\ln\left(\frac{y(1-y_0)}{y_0(1-y)}\right) \quad \text{(Supplementary Equation 2)}$$

directly following from the approximation (Felsenstein, 2019, pp. 75f)

$$y(t) = \frac{y_0}{y_0+(1-y_0)e^{-st}} \quad \text{(Supplementary Equation 3)}$$

where y_0 is the initial allele frequency at the onset of selection and $y(t)$ the frequency at time t.

We then substitute Supplementary Equation 2 into Supplementary Equation 1

$$\begin{aligned}\ln\mathcal{L}(s|r,y) &= 2k\Bigg(\frac{r}{s}\Bigg(-\ln\left(\frac{y\cdot(1-y_0)}{y_0\cdot(1-y)}\right)\\ &\quad+\ln\left(1-y_0\left(1-\frac{y\cdot(1-y_0)}{y_0\cdot(1-y)}\right)\right)\Bigg)\\ &\quad+\ln\left(1-\left(\frac{1-y_0\left(1-\frac{y\cdot(1-y_0)}{y_0\cdot(1-y)}\right)}{\frac{y\cdot(1-y_0)}{y_0\cdot(1-y)}}\right)^{\Delta r/s}\right)\Bigg)\\ &=2k\Bigg(\frac{r}{s}\ln\left(\frac{1-y_0+\frac{y\cdot(1-y_0)}{1-y}}{\frac{y\cdot(1-y_0)}{y_0\cdot(1-y)}}\right)\\ &\quad+\ln\left(1-\left(\frac{1-y_0+\frac{y\cdot(1-y_0)}{1-y}}{\frac{y\cdot(1-y_0)}{y_0\cdot(1-y)}}\right)^{\Delta r/s}\right)\Bigg)\\ &=2k\left(\frac{r}{s}\ln\left(\frac{y_0}{y}\right)+\ln\left(1-\left(\frac{y_0}{y}\right)^{\Delta r/s}\right)\right),\end{aligned} \quad \text{(Supplementary Equation 4)}$$

using the following identity

$$\begin{aligned}\frac{1-y_0+\frac{y\cdot(1-y_0)}{1-y}}{\frac{y\cdot(1-y_0)}{y_0\cdot(1-y)}} &= \frac{y_0(1-y)-(1-y)y_0^2+y(1-y_0)y_0}{y(1-y_0)}\\ &=\frac{y_0-y_0y-y_0^2+y_0^2y+y_0y-y_0^2y}{y(1-y_0)}\\ &=\frac{(1-y_0)y_0}{y(1-y_0)}\\ &=\frac{y_0}{y}.\end{aligned} \quad \text{(Supplementary Equation 5)}$$

In order to maximise, we derive Supplementary Equation 4

$$\begin{aligned}
&\frac{d}{ds}\left(2k\left(\frac{r}{s}\ln\left(\frac{y_0}{y}\right)+\ln\left(1-\left(\frac{y_0}{y}\right)^{\Delta r/s}\right)\right)\right)\\
&=2k\left(-\frac{r}{s^2}\ln\left(\frac{y_0}{y}\right)+\frac{\Delta r}{s^2}\ln\left(\frac{y_0}{y}\right)\frac{\left(\frac{y_0}{y}\right)^{\Delta r/s}}{1-\left(\frac{y_0}{y}\right)^{\Delta r/s}}\right) \qquad \text{(Supplementary Equation 6)}\\
&=2k\frac{\ln\left(\frac{y_0}{y}\right)}{s^2}\left(\Delta r\frac{\left(\frac{y_0}{y}\right)^{\Delta r/s}}{1-\left(\frac{y_0}{y}\right)^{\Delta r/s}}-r\right),
\end{aligned}$$

and determine the optimum by equating to zero

$$\begin{aligned}
&0=2k\frac{\ln\left(\frac{y_0}{y}\right)}{s^2}\left(\Delta r\frac{\left(\frac{y_0}{y}\right)^{\Delta r/s}}{1-\left(\frac{y_0}{y}\right)^{\Delta r/s}}-r\right)\\
\iff &0=\Delta r\frac{\left(\frac{y_0}{y}\right)^{\Delta r/s}}{1-\left(\frac{y_0}{y}\right)^{\Delta r/s}}-r\\
\iff &\Delta r\left(\frac{y_0}{y}\right)^{\Delta r/s}=r\left(1-\left(\frac{y_0}{y}\right)^{\Delta r/s}\right) \qquad \text{(Supplementary Equation 7)}\\
\iff &(\Delta r+r)\left(\frac{y_0}{y}\right)^{\Delta r/s}=r\\
\iff &\left(\frac{y_0}{y}\right)^{\Delta r/s}=\frac{r}{\Delta r+r}\\
\iff &\frac{\Delta r}{s}\ln\left(\frac{y_0}{y}\right)=\ln\left(\frac{r}{\Delta r+r}\right)
\end{aligned}$$

to finally obtain

$$\hat{s}=\frac{\Delta r}{\ln\left(\frac{r}{\Delta r+r}\right)}\cdot\ln\left(\frac{y_0}{y}\right). \qquad \text{(Supplementary Equation 8)}$$

Filtering blocks—choice of thresholds

In order to determine filter thresholds that minimise false positive calls while achieving good accuracy, we considered all haploblocks that overlap the midpoint of the simulated chromosome segment detected in four arbitrarily chosen simulations per simulated selection coefficient generated as part of Figure S9, at time points where the selected allele has frequencies 0% and 60%. Note that the former effectively corresponds to a neutral simulation.

For various combinations of thresholds, the subset of these blocks that do not have the selected allele are counted as false positives if they pass the filters, and as true negatives if they do not; while those

that do have the selected allele are counted as true positives if they pass the filters, and as false negatives if they do not. We note that unlike in the confusion matrices for example shown in Figures S5-S8, a low true positive rate is not a problem, as in principle a single block is enough to infer selection at the midpoint. The main criteria for the choice of thresholds is therefore to minimise false positives, followed by the accuracy of the point estimation of the selection coefficient.

In a first step, we set the threshold on $\hat{t_1}$ to 1, effectively disabling the filter defined by Equations 7-12, and try various thresholds on $\hat{t_2}$ as defined by Equation 17. The results are summarised in Table S1, and show that on its own no threshold is enough to fully avoid false positives. This filtering step is intended primarily to catch long blocks with few haplotypes, expected under recent common ancestry, and we therefore opt to continue with a threshold of 1%. As shown in Figures S3 and S4, this is indeed enough to filter long blocks containing only few haplotypes.

In a second step, we look for the strongest threshold on $\hat{t_1}$ that leads to no false yet some true positives when applied after the previous filtering step with threshold of 1%. As can be seen in Table S2, only thresholds of 1% and the more stringent adaptive threshold defined by Equations 10-12 satisfy these conditions, and we therefore chose the latter. The adaptive threshold on $\hat{t_1}$ is parametrised by a minimal detectable selection coefficient s_{min}. The underlying quantile is at least 1%, but not lower than 0.01% to avoid comparing very small numbers. The reasoning is that in relation to selection the effect of drift, which is not modelled as part of our sweep model, is stronger for low frequency alleles, and such an adaptive threshold therefore represents an additional safeguard against false positives.

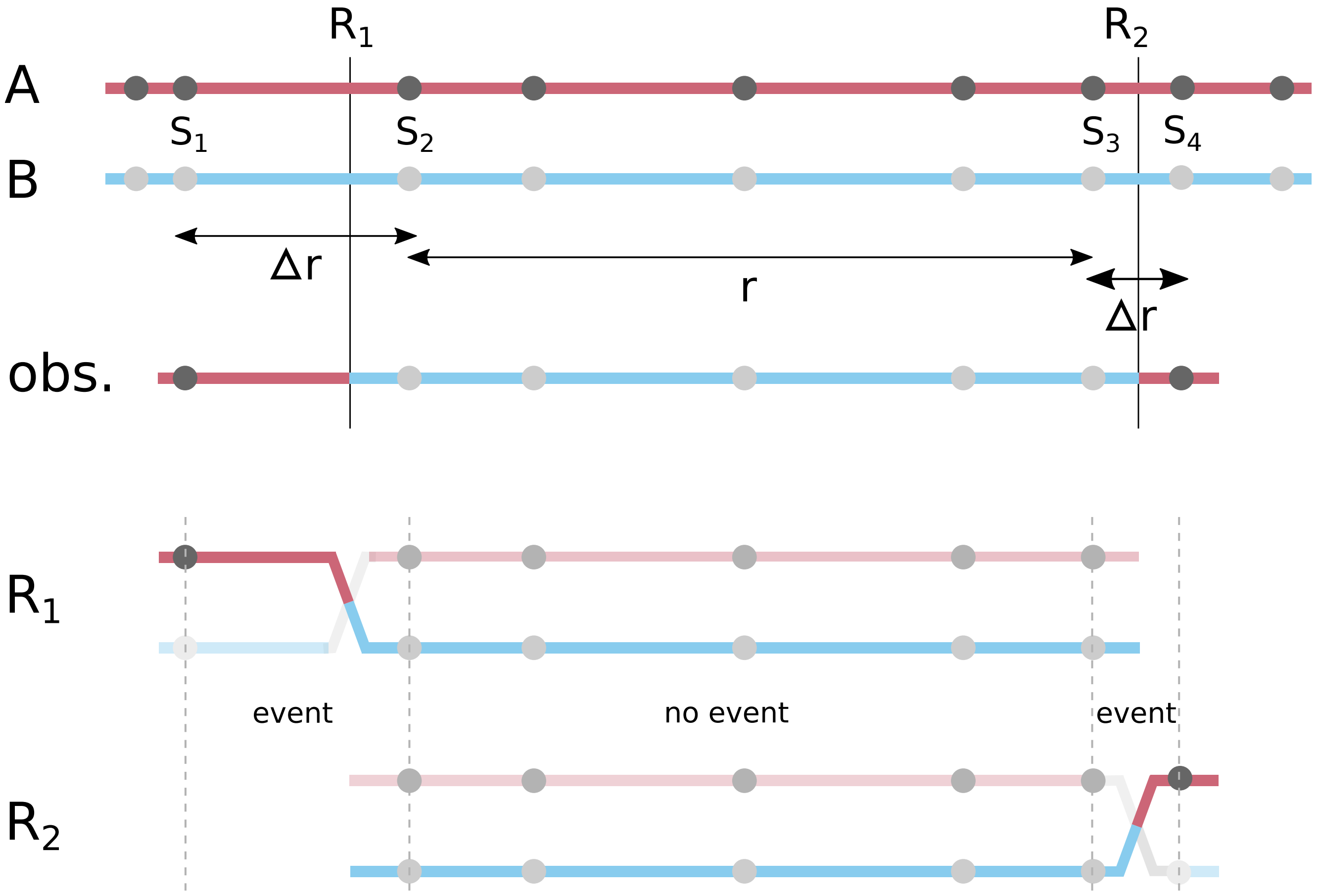

FIG. S1. *Probability of a haplotype in a haplotype block.* The red and blue lines indicate haplotypes A and B respectively and dots indicate SNPs. SNPs S_1, S_2, S_3, S_4 define the regions that lie between or contain the breakpoints. The probability of observing the haplotype denoted by 'obs.' (observed) is the product of two independent recombination events R_1 and R_2, i.e. the probability of no event in a region with recombination fraction r times the probability of one event in a region with recombination fraction Δr.

Table S1. *Haploblocks and inferred selection coefficients at different filter thresholds.* We count all haploblocks that overlap the midpoint of the simulated chromosome segment detected in four arbitrarily chosen simulations per simulated selection coefficient generated as part of Figure S9, at time points where the selected allele has frequencies 0% and 60%. For different combinations of thresholds on $\hat{t_1}$ and $\hat{t_2}$, HB that do not have the selected allele are counted as false positives (FP) if they pass the filters, and as true negatives (TN) if they do not, and if HB have the selected allele as true positives (TP) if they pass the filters, and as false negatives (FN) if they do not. False positive rate (FPR) is computed as $\frac{FP}{FP+TN}$, true positive rate (TPR) as $\frac{TP}{TP+FN}$. *Abbreviations:* simulated (sim.), threshold (thresh.), maximal (max.)

sim. s	$\hat{t_1}$ thresh.	$\hat{t_2}$ thresh.	TP	FN	TN	FP	FPR	TPR	max. $\hat{s}$
0.0075	1	0	0	21933	31998	0	0	0	0
0.0075	1	1E-04	835	20787	31851	147	0.005	0.039	0.0267
0.0075	1	0.001	3665	16697	30182	1816	0.057	0.18	0.0378
0.0075	1	0.01	7377	12548	25858	6140	0.192	0.37	0.0531
0.0075	1	0.1	12598	7294	18307	13691	0.428	0.633	0.132
0.0075	1	1	21933	0	0	31998	1	1	0.3689
0.01	1	0	0	21985	27126	0	0	0	0
0.01	1	1E-04	1034	20207	26969	157	0.006	0.049	0.0379
0.01	1	0.001	4216	16367	25985	1141	0.042	0.205	0.0462
0.01	1	0.01	8572	11547	22449	4677	0.172	0.426	0.077
0.01	1	0.1	13451	7222	15971	11155	0.411	0.651	0.1811
0.01	1	1	21985	0	0	27126	1	1	0.3912
0.02	1	0	0	29671	31450	0	0	0	0
0.02	1	1E-04	6507	21526	31316	134	0.004	0.232	0.0272
0.02	1	0.001	11121	16886	30074	1376	0.044	0.397	0.0498
0.02	1	0.01	16267	12132	26187	5263	0.167	0.573	0.0628
0.02	1	0.1	21078	7305	18727	12723	0.405	0.743	0.2242
0.02	1	1	29671	0	0	31450	1	1	0.344
0.05	1	0	0	55389	31333	0	0	0	0
0.05	1	1E-04	32329	19852	31209	124	0.004	0.62	0.0623
0.05	1	0.001	38104	14940	29840	1493	0.048	0.718	0.0629
0.05	1	0.01	42987	10970	25950	5383	0.172	0.797	0.0694
0.05	1	0.1	47567	6948	18590	12743	0.407	0.873	0.1518
0.05	1	1	55389	0	0	31333	1	1	0.3901

Table S2. *Haploblocks and inferred selection coefficients at different filter thresholds.* See caption of Table S1. *Abbreviations:* adaptive (adapt.)

sim. s	$\hat{t}_1$ thresh.	$\hat{t}_2$ thresh.	TP	FN	TN	FP	FPR	TPR	max. $\hat{s}$
0.0075	0.01	0.01	316	21615	31998	0	0	0.014	0.0066
0.0075	*adapt.*	0.01	143	21790	31998	0	0	0.007	0.0066
0.01	0.01	0.01	933	20864	27126	0	0	0.043	0.0121
0.01	*adapt.*	0.01	652	21163	27126	0	0	0.03	0.0121
0.02	1E-04	0.01	1693	27865	31450	0	0	0.057	0.0156
0.02	0.001	0.01	4579	25011	31450	0	0	0.155	0.0175
0.02	0.01	0.01	8554	20829	31450	0	0	0.291	0.0189
0.02	*adapt.*	0.01	7188	22018	31450	0	0	0.246	0.0189
0.05	1E-04	0.01	28352	26549	31333	0	0	0.516	0.0317
0.05	0.001	0.01	32675	21901	31333	0	0	0.599	0.0346
0.05	0.01	0.01	35622	18822	31333	0	0	0.654	0.0625
0.05	*adapt.*	0.01	35184	19155	31333	0	0	0.647	0.0625

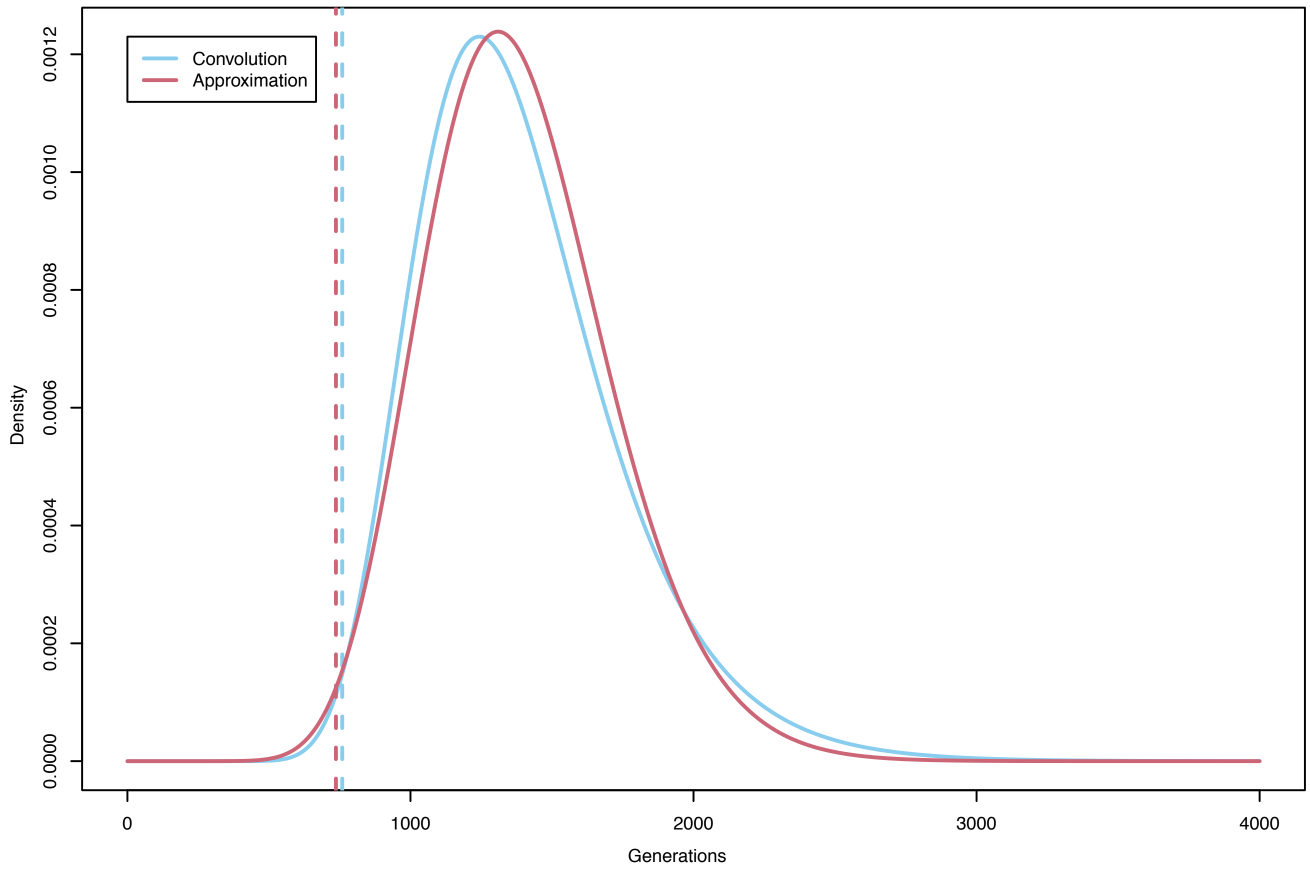

FIG. S2. *Exact and approximated* t_{MRCA} *of a haplotype block.* The distributions show the probability density function of the t_{MRCA} (convolution) and its approximation by a single gamma distribution for an effective population size of $N_e = 10{,}000$, recombination fraction $r = 0.002$, and frequency $k = 50$. Vertical dashed lines indicate the corresponding first percentiles.

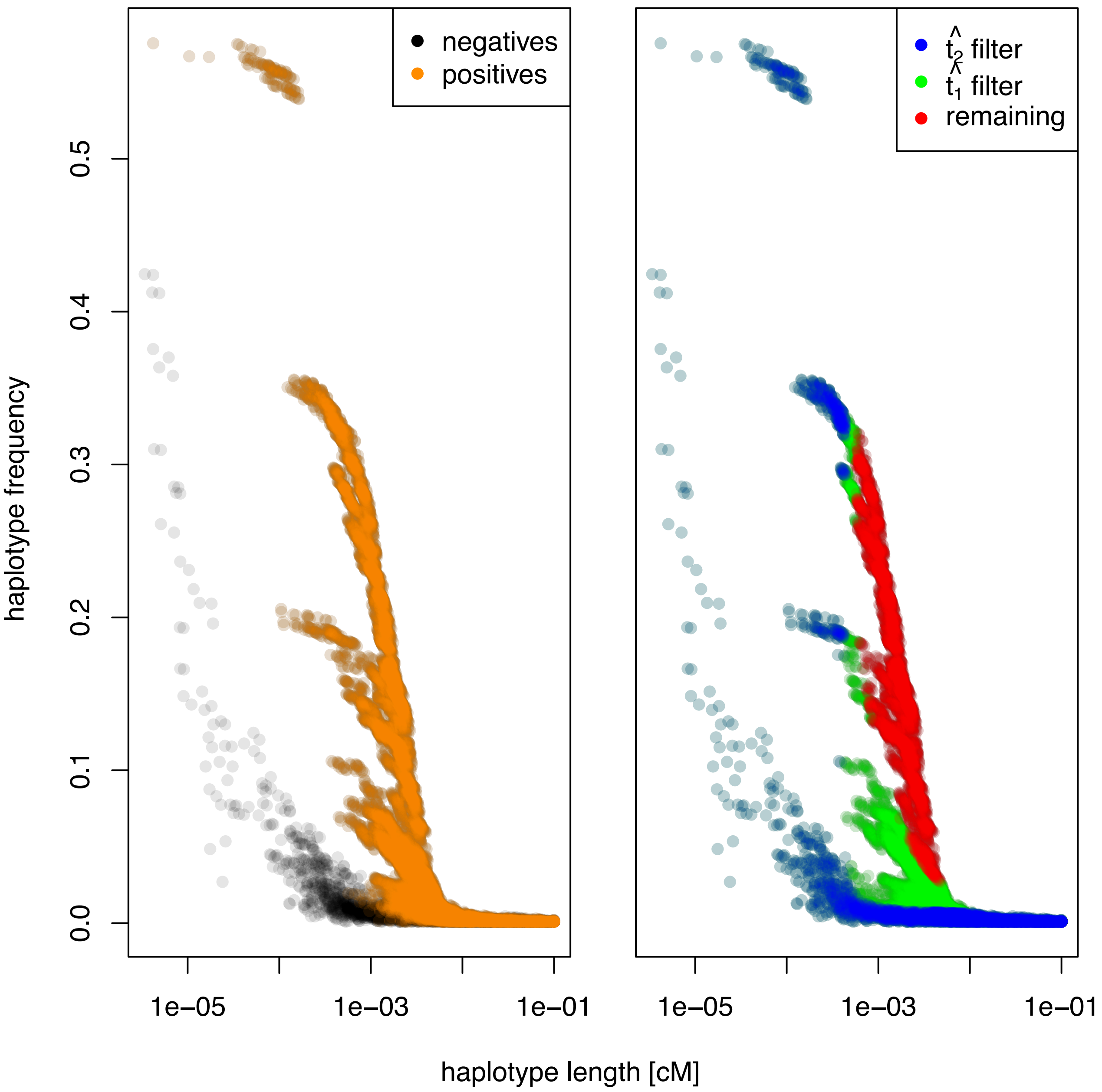

FIG. S3. *Length and frequency of haploblocks found in data simulated with* $s = 0.05$. We show length and frequency of all haploblocks that overlap the midpoint of the simulated chromosome segment detected in an arbitrarily chosen simulation with selection coefficient $s = 0.05$ generated as part of Figure S9, at time points where the selected allele has frequencies 0% and 60%. These are a subset of the blocks listed in the last row of Table S2. Filter thresholds are 1% for the $\hat{t_2}$ filter, and the adaptive one for the $\hat{t_1}$ filter.

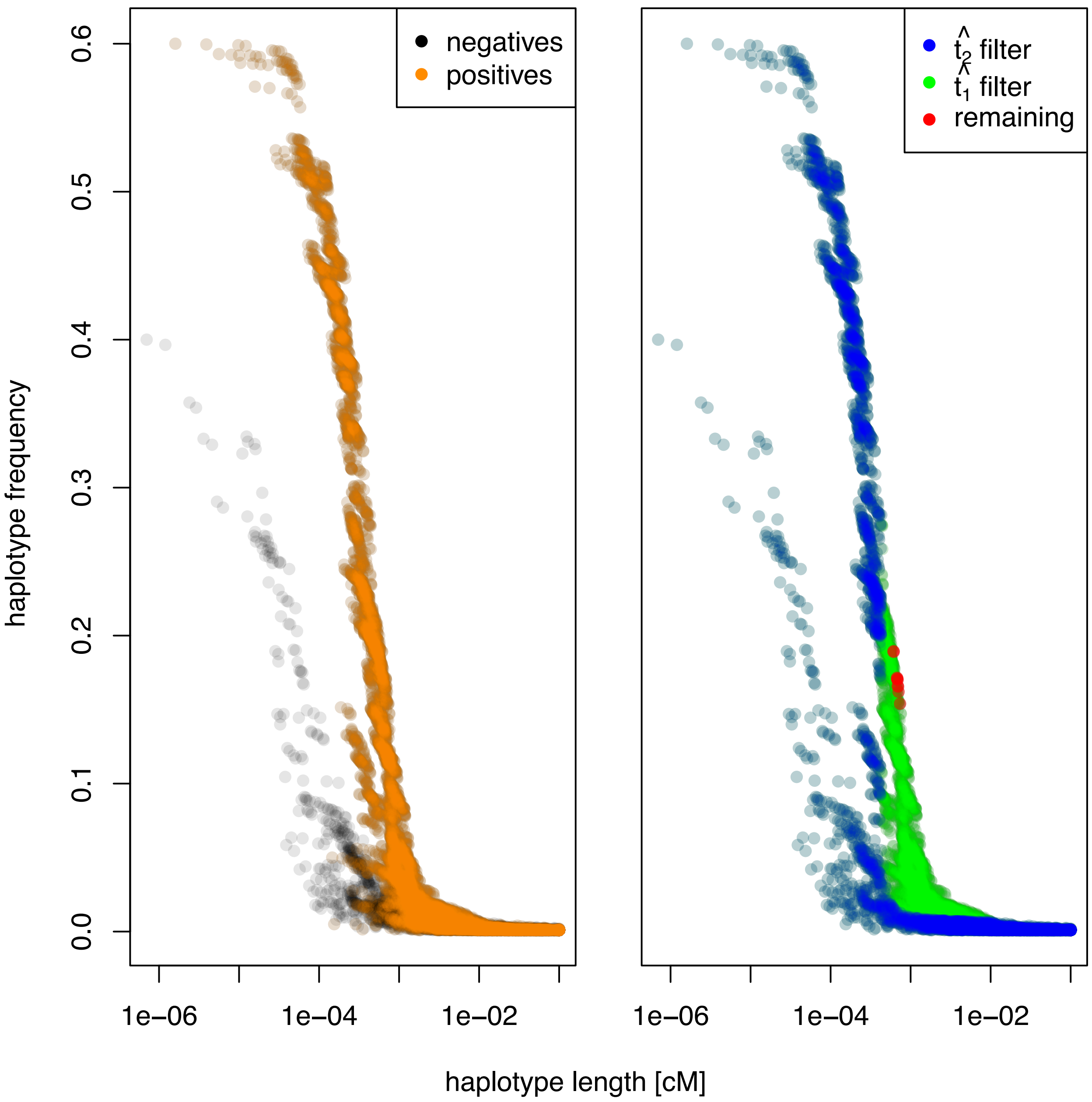

FIG. S4. *Length and frequency of haploblocks found in data simulated with* $s = 0.0075$. See caption of Figure S3 for details. These are a subset of the blocks listed in the second data row of Table S2.

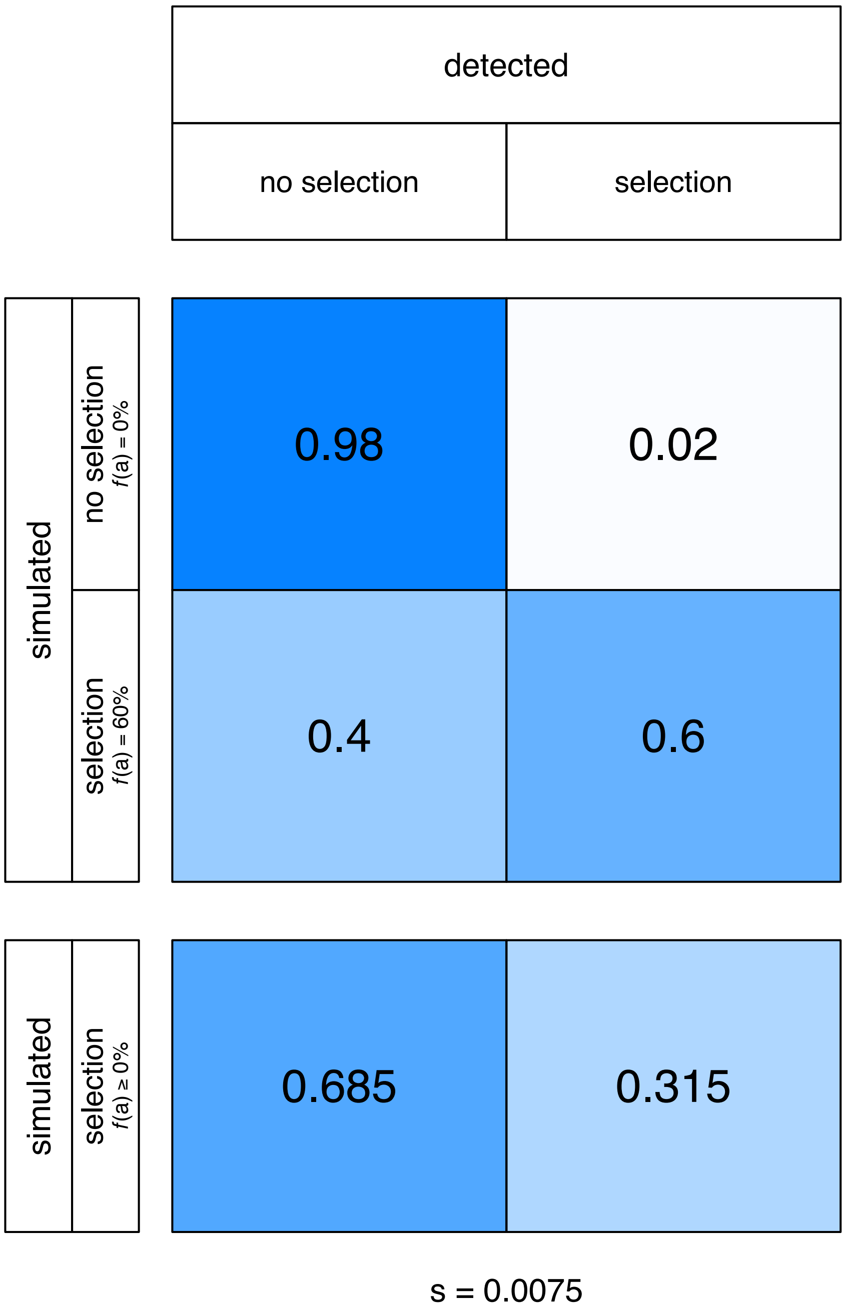

FIG. S5. *Inference accuracy of the presence/absence of selection for simulations with* $s = 0.0075$. For the 50 simulations with $s = 0.0075$ from Figure 1a, we show proportions of the confusion matrix. We count each simulation once, and infer selection if at least one block that carries the selected allele covers the midpoint, i.e. the locus simulated with selection. We show true positive and false negative proportions both for time points where the frequency $f(a)$ of the beneficial allele a is at 60% (middle row), and jointly for the 12 frequencies above 0% (third row; frequencies 2%, 5%, 10%, 20%, 30%, 40%, 50%, 60%, 70%, 80%, 90%, 100%). See Materials and Methods for details on the simulations.

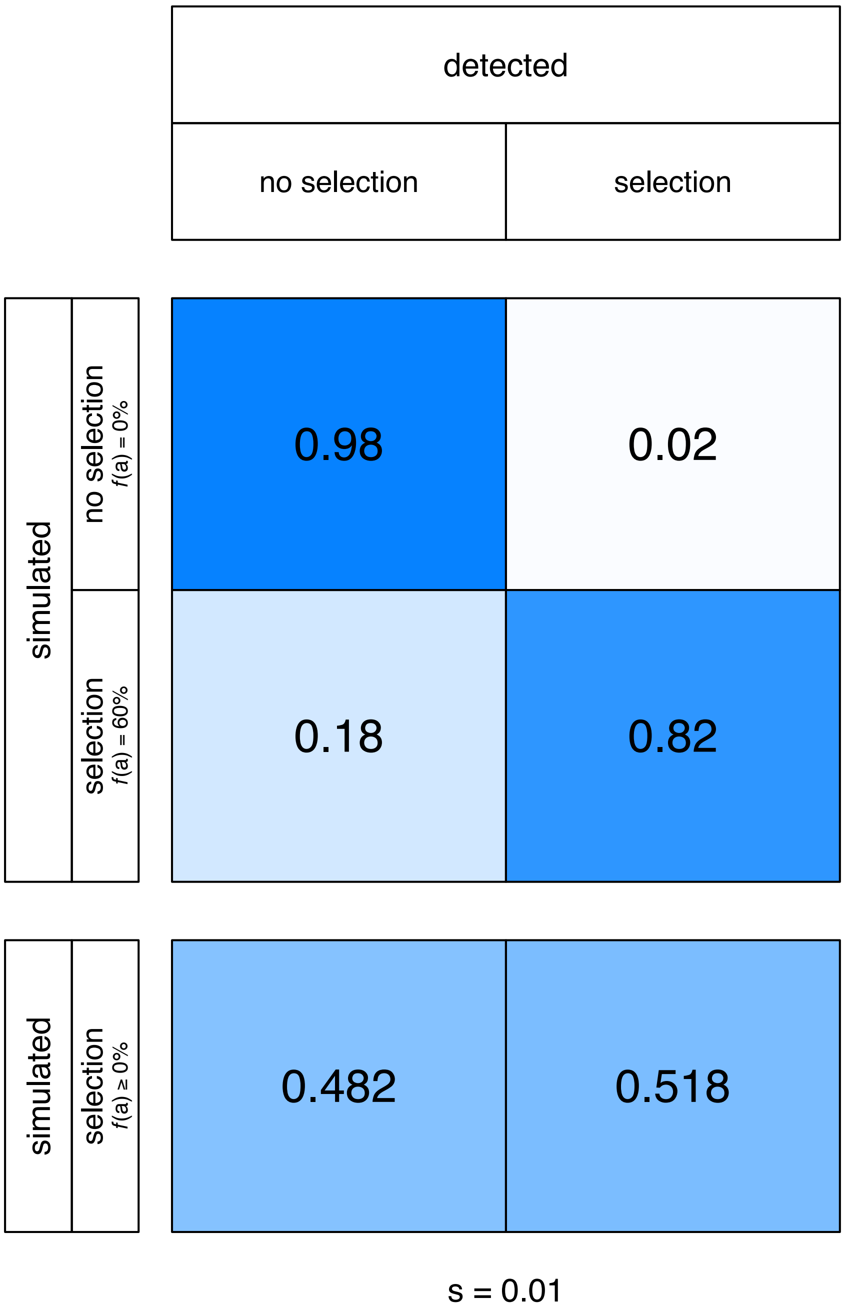

FIG. S6. *Inference accuracy of the presence/absence of selection for simulations with* $s = 0.01$. See caption of Figure S5 for details.

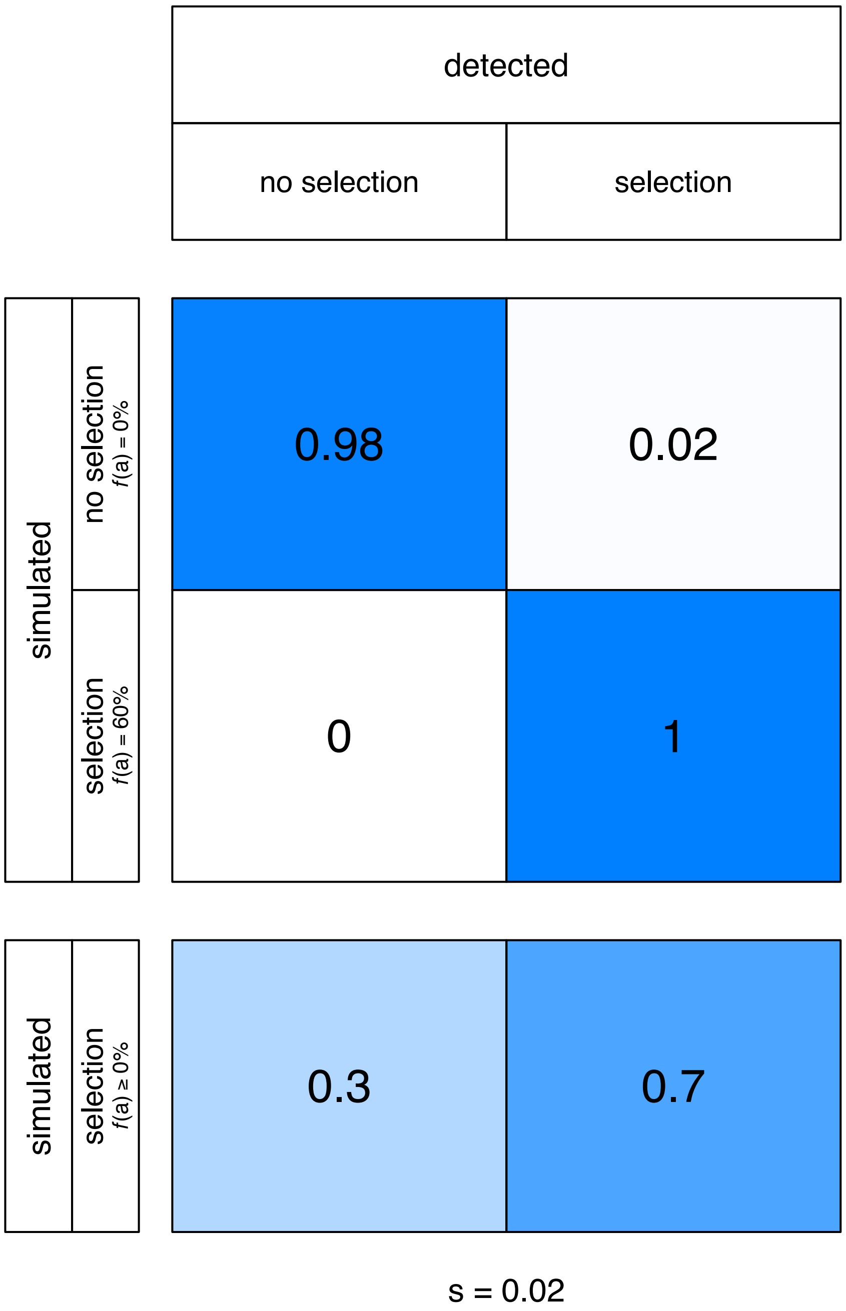

FIG. S7. *Inference accuracy of the presence/absence of selection for simulations with* $s = 0.02$. See caption of Figure S5 for details.

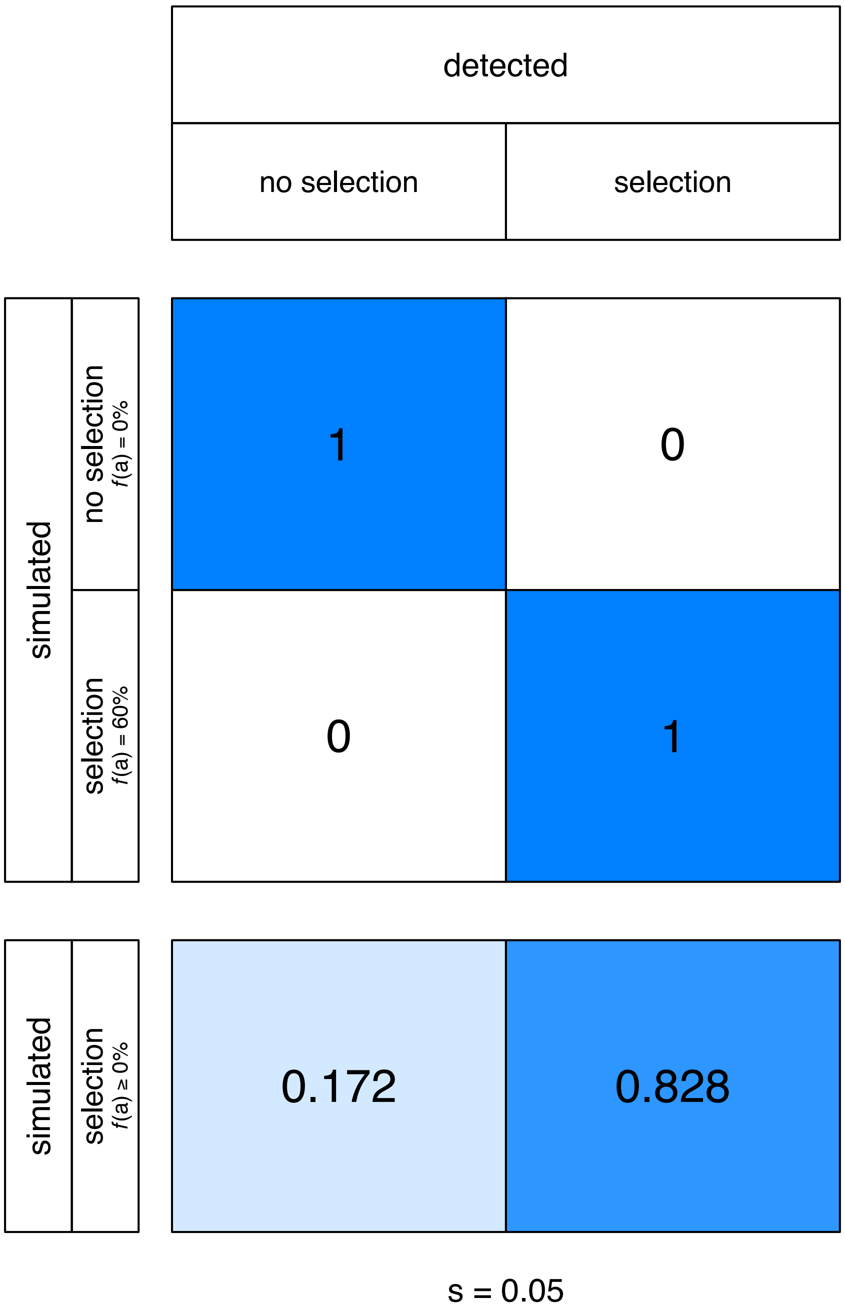

FIG. S8. *Inference accuracy of the presence/absence of selection for simulations with* $s = 0.05$. See caption of Figure S5 for details.

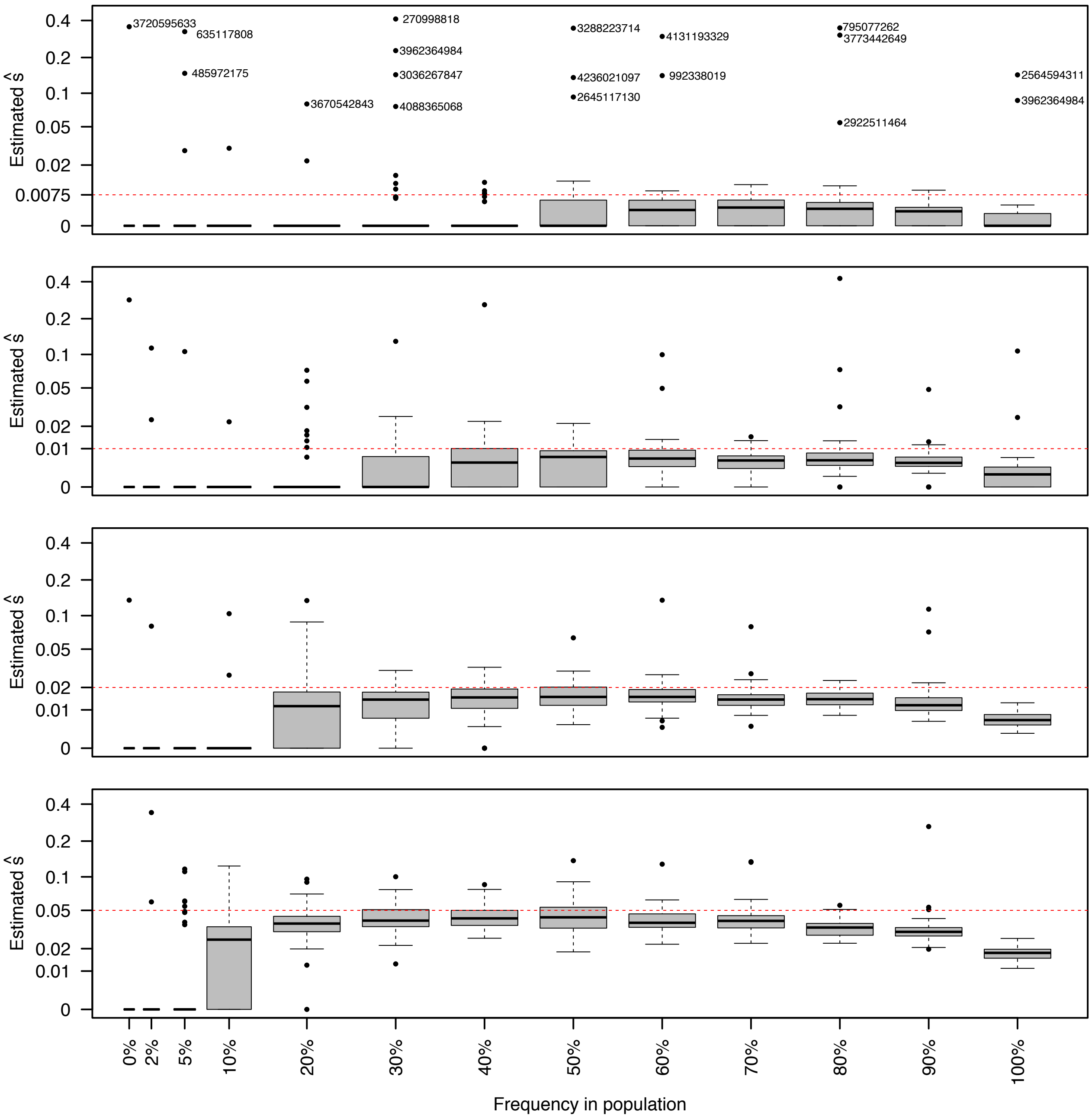

FIG. S9. *Distributions of estimated selection coefficients summarised in Figure 1a.* The y-axis is logarithmic, and the dashed red line indicates the simulated selection coefficient. The upper panel additionally shows the simulation ID for outliers, illustrating that no single simulation produced outliers over all frequencies.

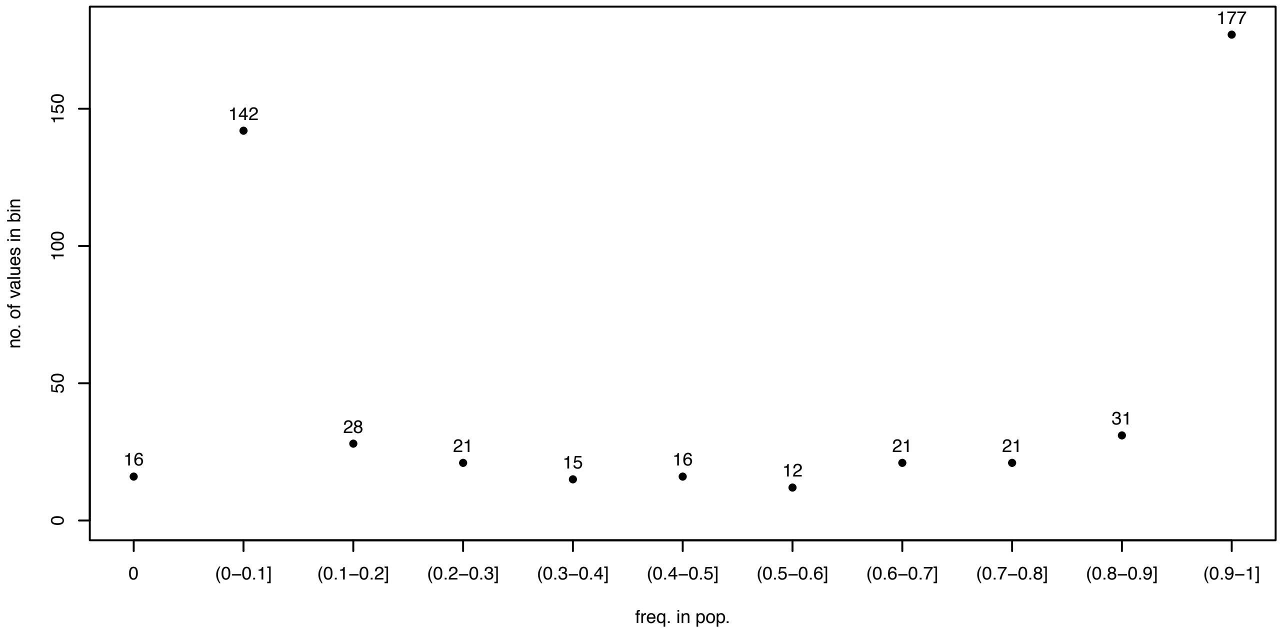

FIG. S10. *Number of simulations in frequency bins of Figure 1b.*

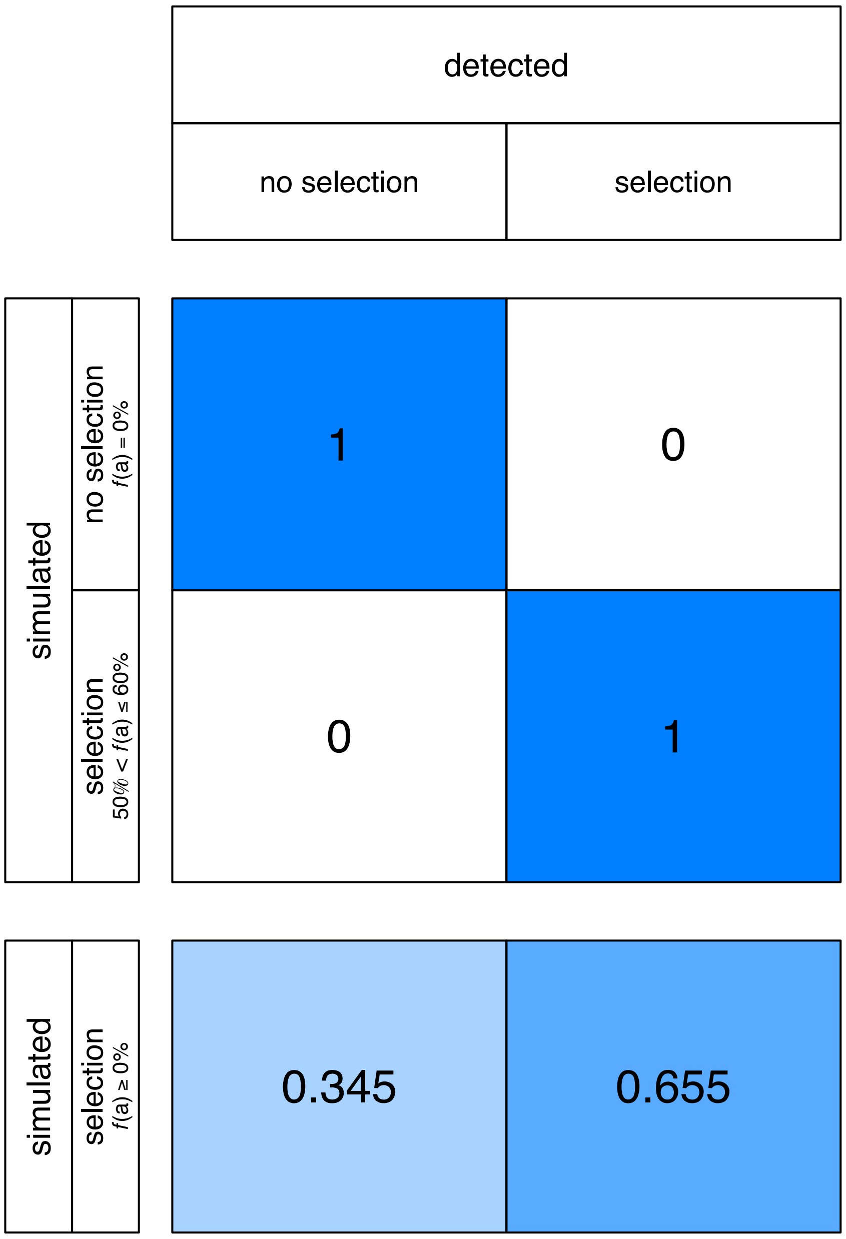

FIG. S11. *Inference accuracy of the presence/absence of selection for simulations with* $s = 0.03$ *under an Out-of-Africa model.* For the simulations from Figure 1b, we show proportions of the confusion matrix. We count each simulation once, and infer selection if at least one block that carries the selected allele covers the midpoint, i.e. the locus simulated with selection. We show true positive and false negative proportions both for the simulations where the selected allele frequency reached between 60% and 70% (middle row), and jointly for the 10 frequency ranges above 0% detailed in Figure S10 (third row). See Materials and Methods for details on the simulations.

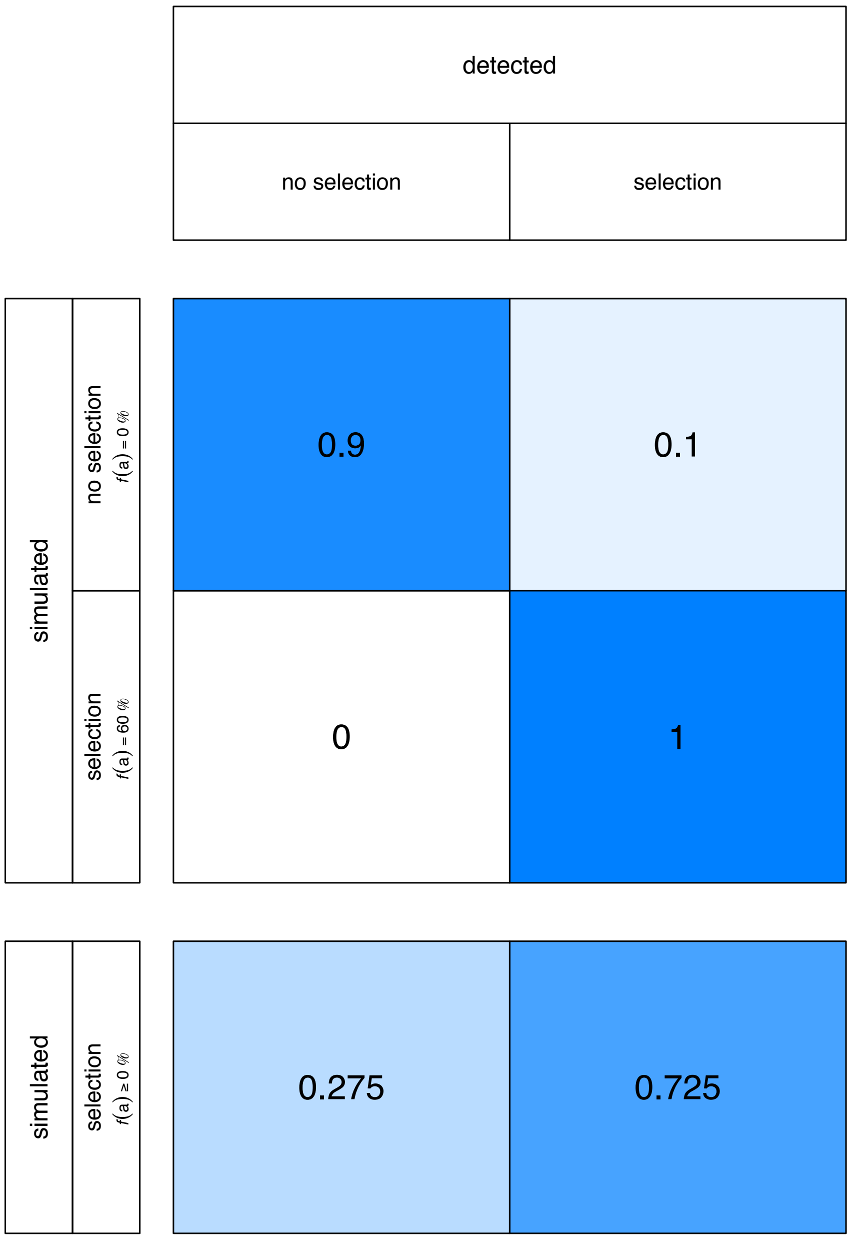

FIG. S12. *Inference accuracy of the presence/absence of selection for simulations with* $s = 0.03$ *under a demographic model with bottleneck.* We show proportions of the confusion matrix for 20 simulations. We count each simulation once, and infer selection if at least one block that carries the selected allele covers the midpoint, i.e. the locus simulated with selection. We show true positive and false negative proportions both for time points where the frequency $f(a)$ of the beneficial allele a is at 60% (middle row), and jointly for the 12 frequencies above 0% (third row; frequencies 2%, 5%, 10%, 20%, 30%, 40%, 50%, 60%, 70%, 80%, 90%, 100%). See Materials and Methods for details on the simulations.

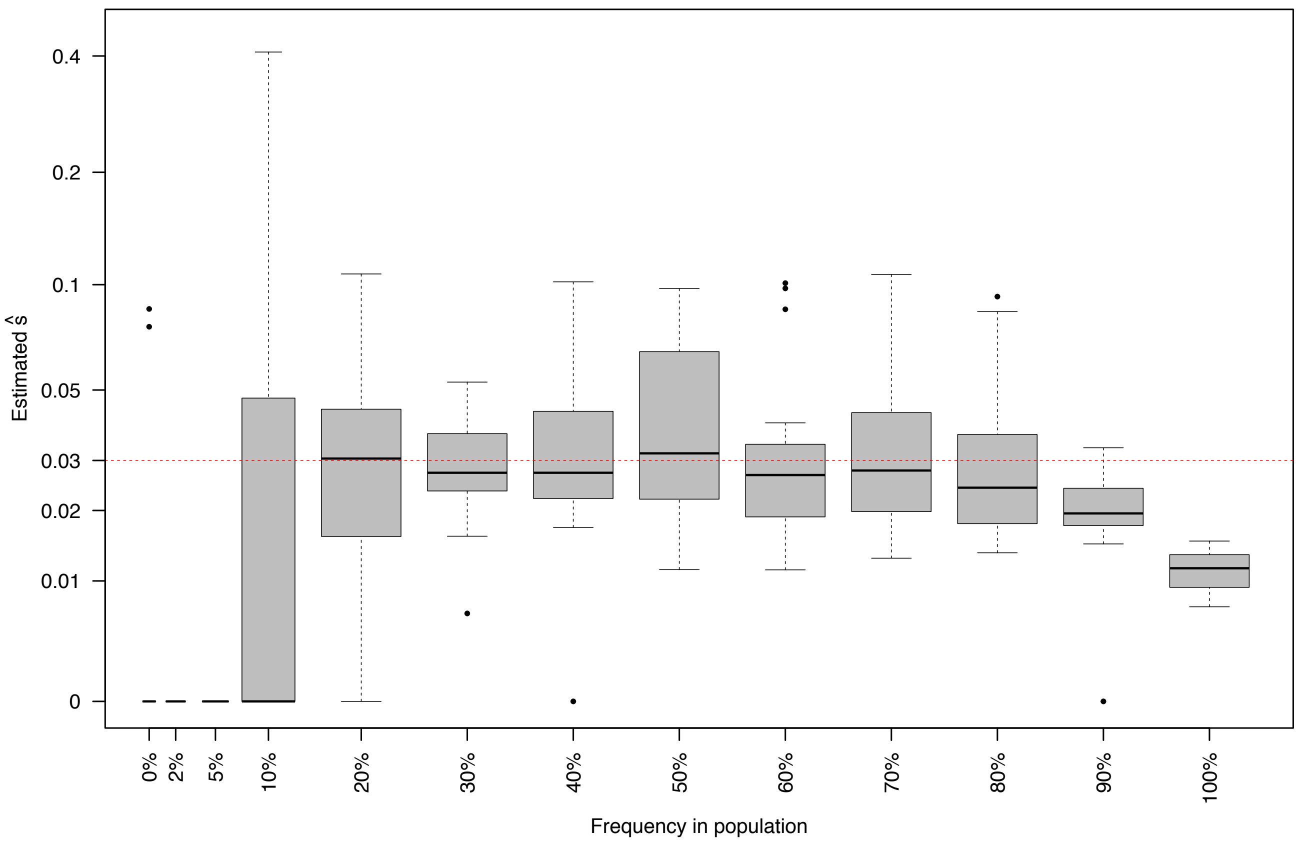

FIG. S13. *Distributions of estimated selection coefficients summarised in Figure S12.*

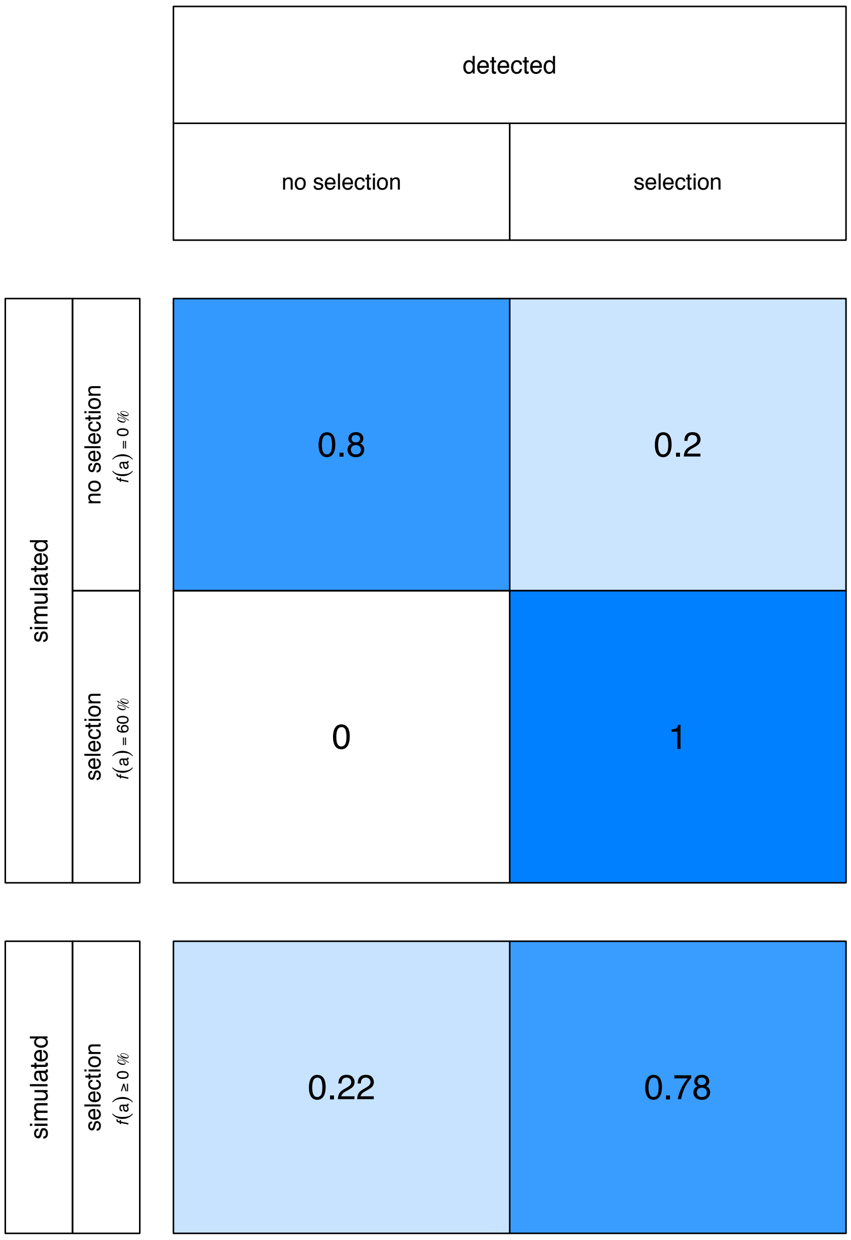

FIG. S14. *Inference accuracy of the presence/absence of selection for simulations with* $s = 0.03$ *under a demographic model with migration.* See caption of Figure S12 for details.

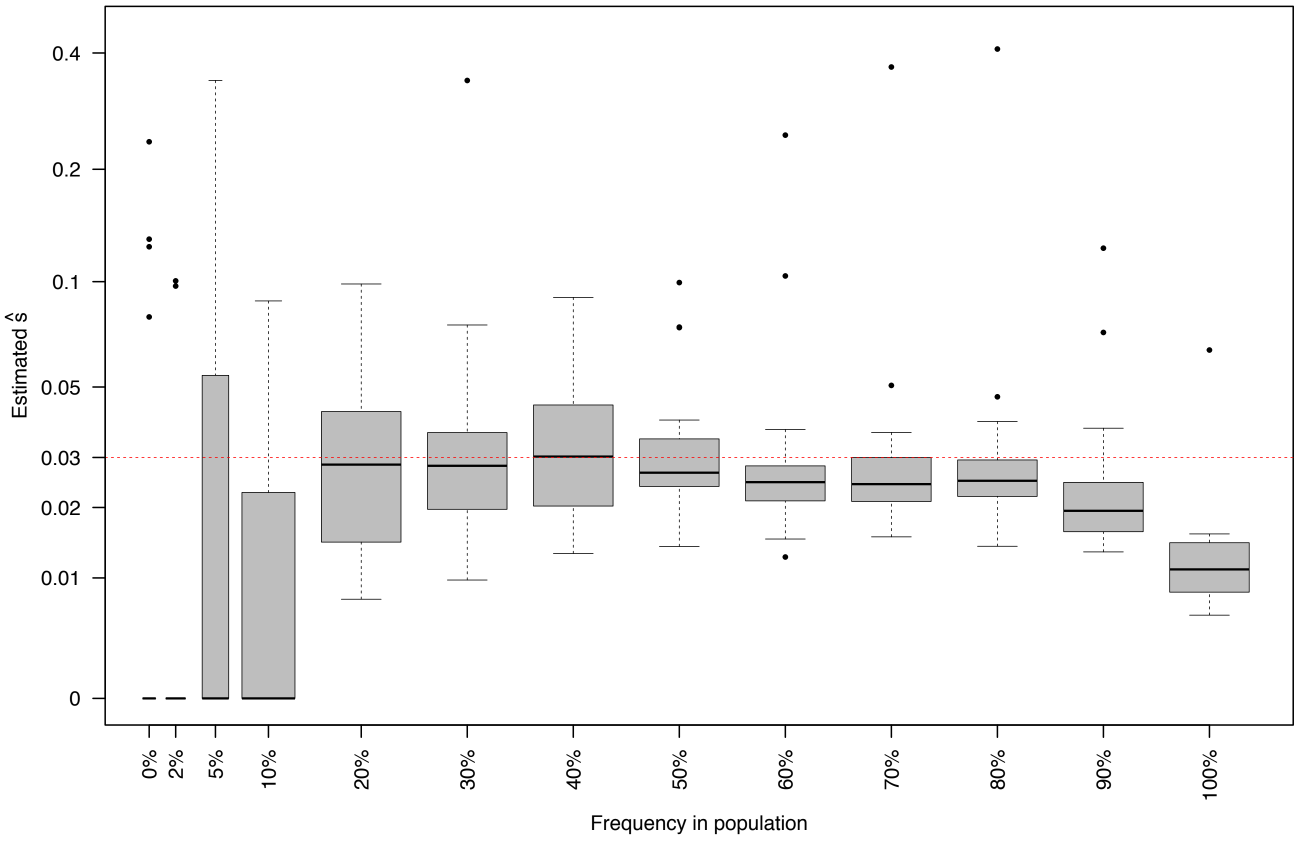

FIG. S15. *Distributions of estimated selection coefficients summarised in Figure S14.*

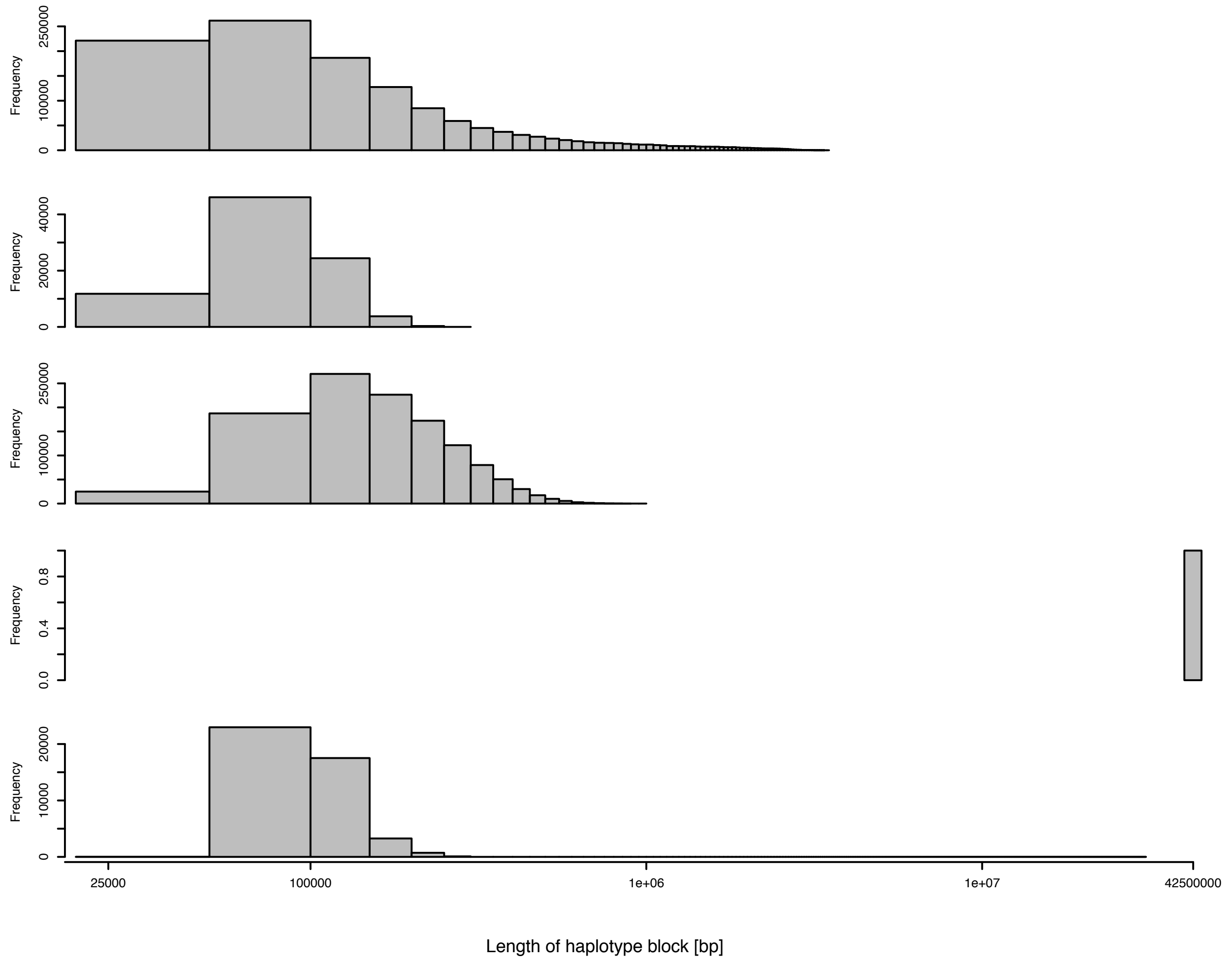

FIG. S16. *Length distribution of HBs found in chromosome 2 of the UK Biobank genotype array data, and in neutral simulations of chromosome 2.* The first row shows a total of 1,447,947 blocks that passed both filtering steps and do not overlap large gaps in the assembly in the UK Biobank. Rows two to four show results of neutral simulations, where all blocks passed both filtering steps and therefore represent false positives: first, 88,906 blocks in a simulation with constant population size downsampled to ∼48k SNPs; second, 1,252,333 blocks in a simulation with bottleneck and exponential population growth downsampled to ∼48k SNPs; third, results for the constant population size simulation downsampled to ∼800k SNPs; and lastly results for the simulation with bottleneck and exponential population growth without downsampling (721,189 SNPs). See Materials and Methods for details on the simulations and downsampling of SNPs.

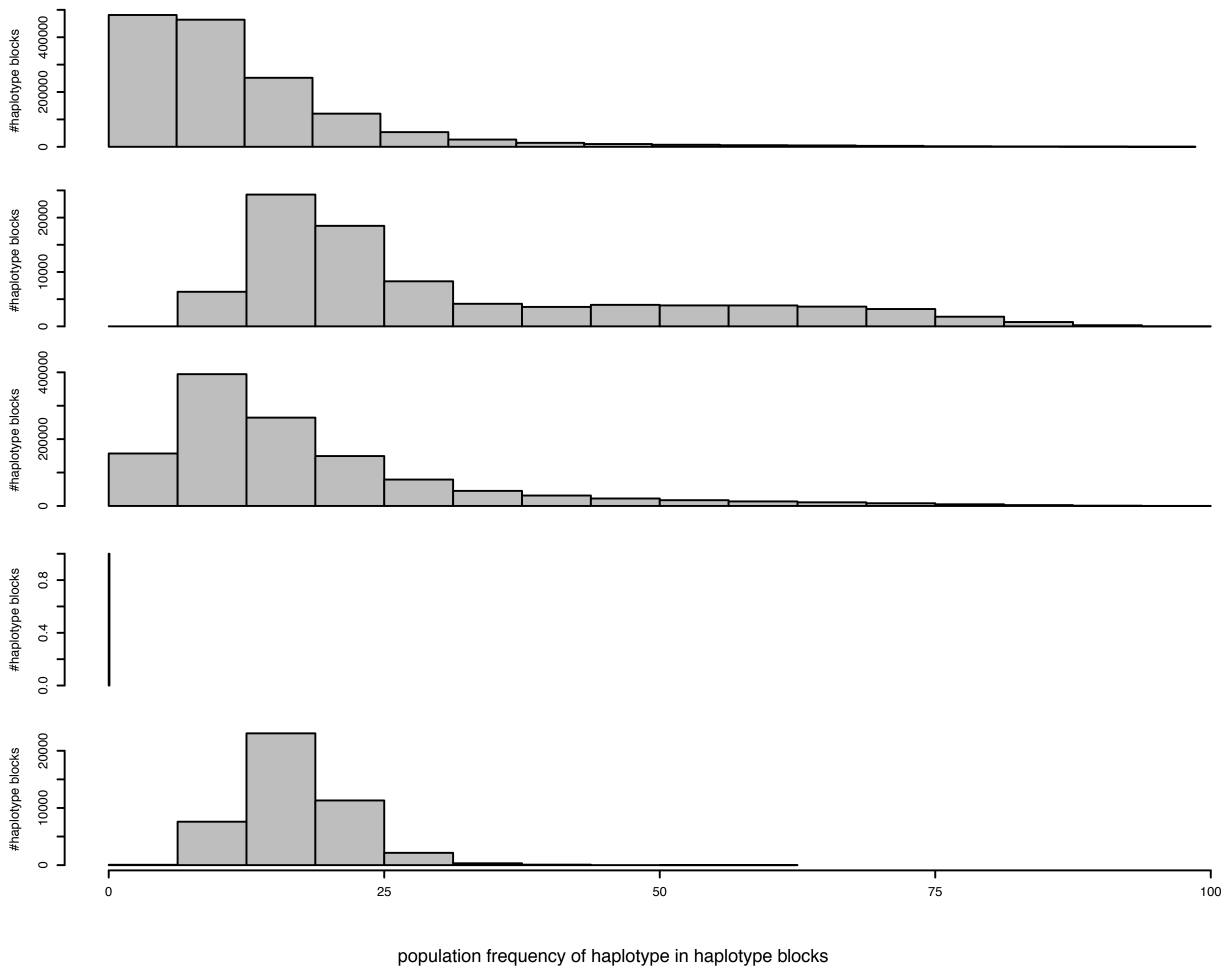

FIG. S17. *Distribution of number of haplotypes in a HB for HBs found in chromosome 2 of the UK Biobank genotype array data, and in neutral simulations of chromosome 2.* The number of haplotypes in a HB on the x-axis is shown as frequency with respect to the full number of chromosomes in the sample, as this number differs between UK Biobank data (811,246 in the first row) and simulations (8,000 for rows 2-5). See caption of Figure S16 for further details.

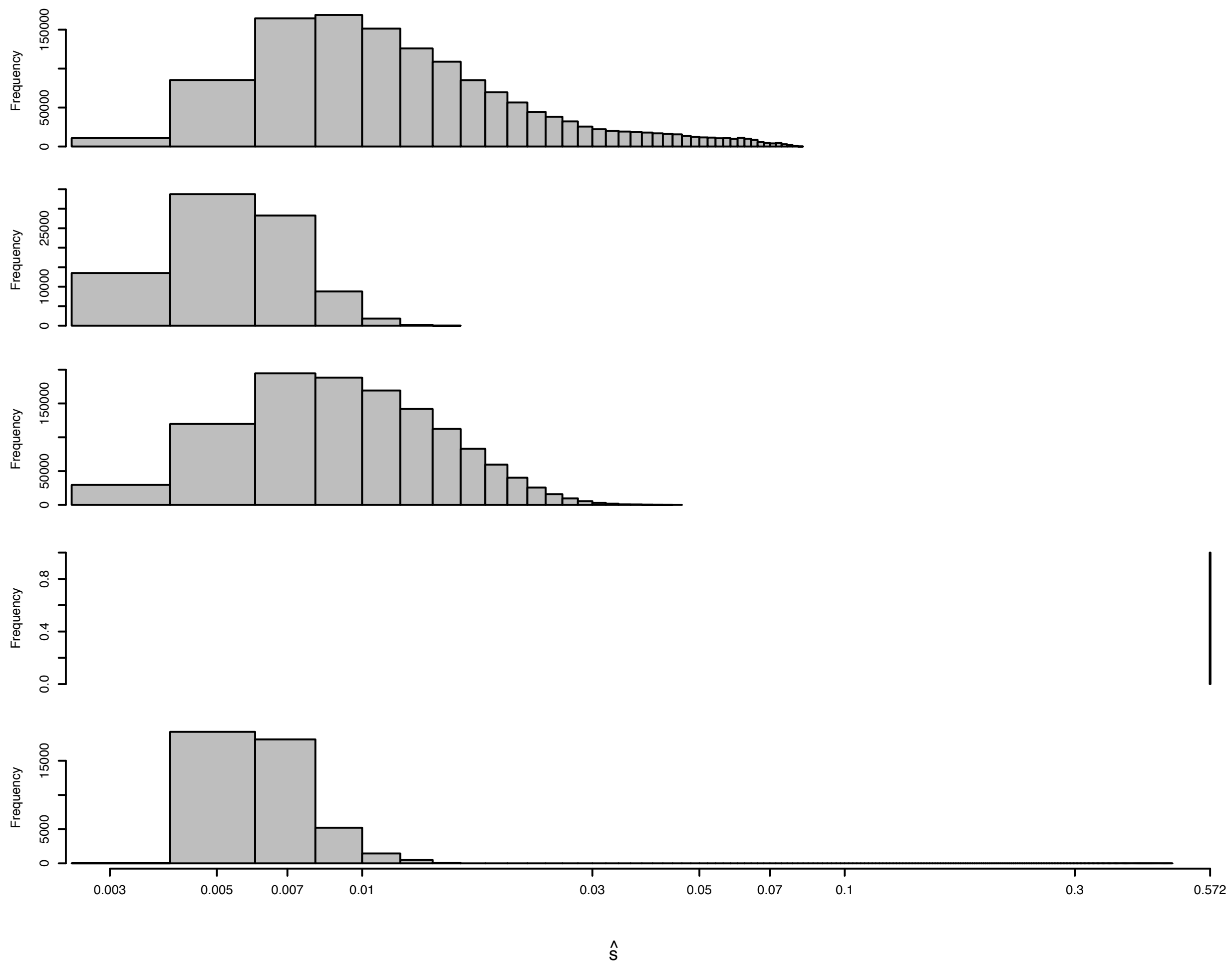

FIG. S18. *Distribution of inferred selection coefficients for HBs found in chromosome 2 of the UK Biobank genotype array data, and in neutral simulations of chromosome 2.* See caption of Figure S16 for details.

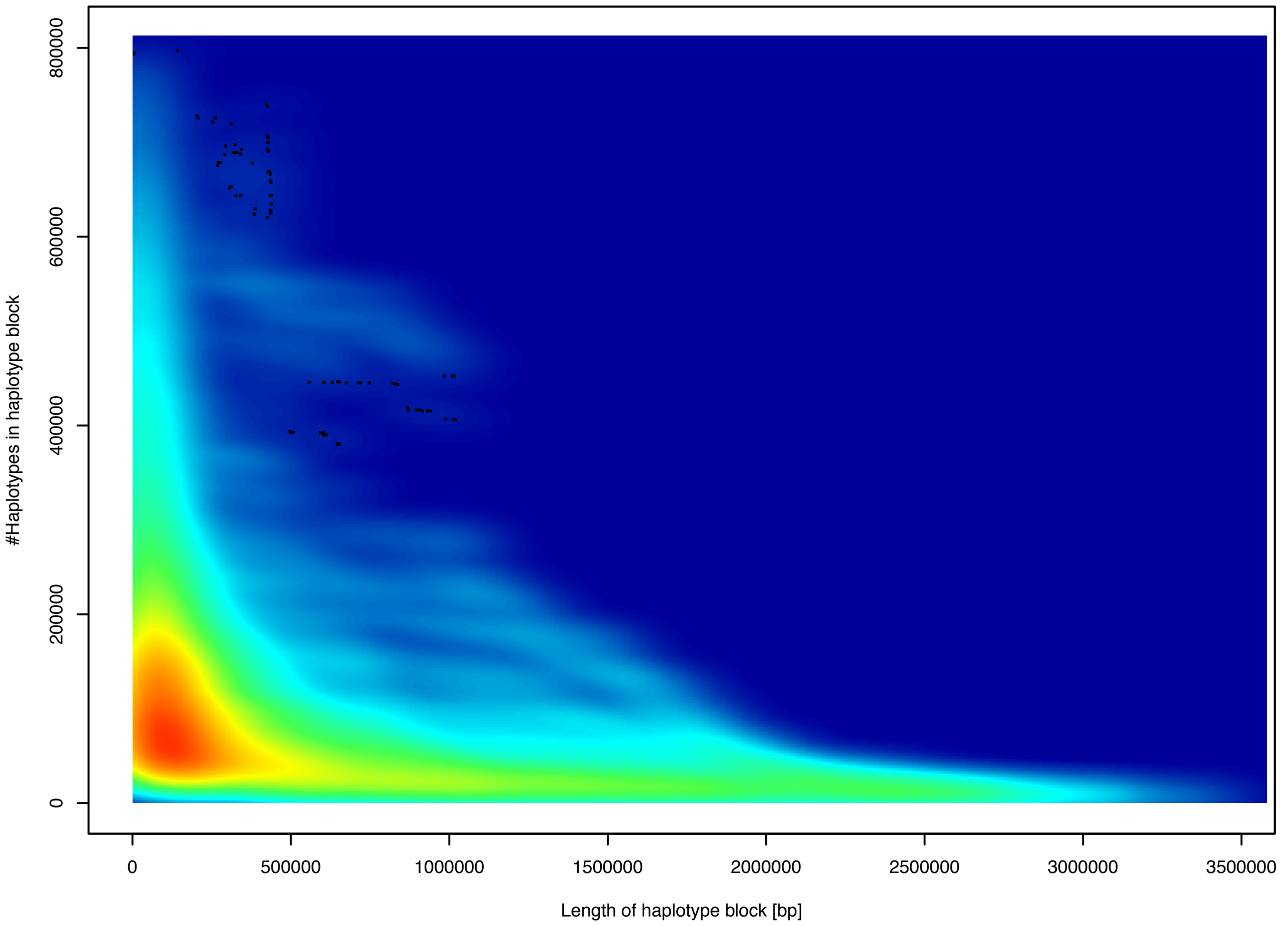

FIG. S19. *Heatmap of joint distribution of haploblock length and number of haplotypes in a HB for HBs found in chromosome 2 of the UK Biobank.* See caption of Figure S16 for details.

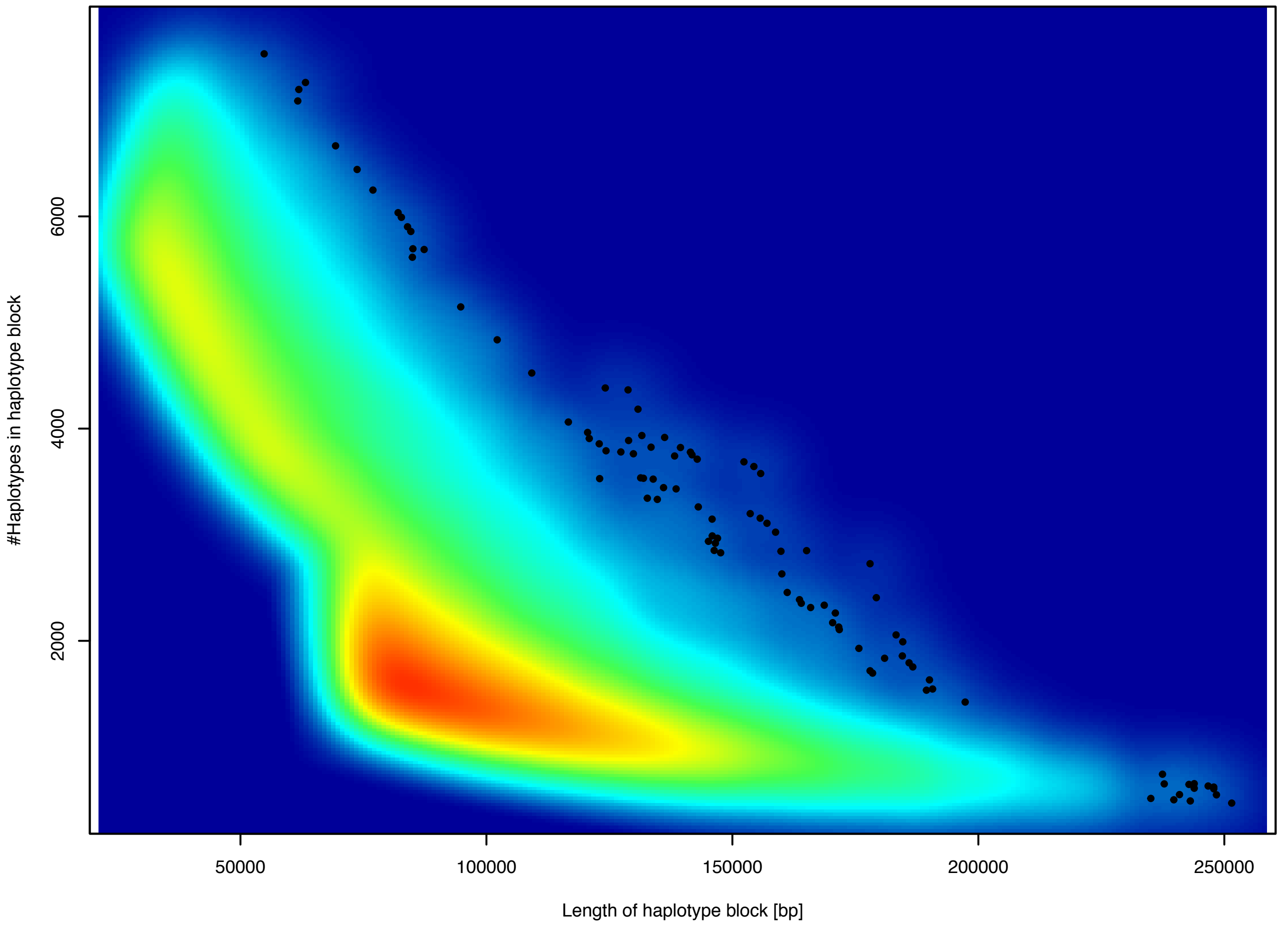

FIG. S20. *Heatmap of joint distribution of haploblock length and number of haplotypes in a HB for HBs found in a neutral simulation of chromosome 2 with constant pppulation size and downsampled to ∼48k SNPs.* See caption of Figure S16 for details.

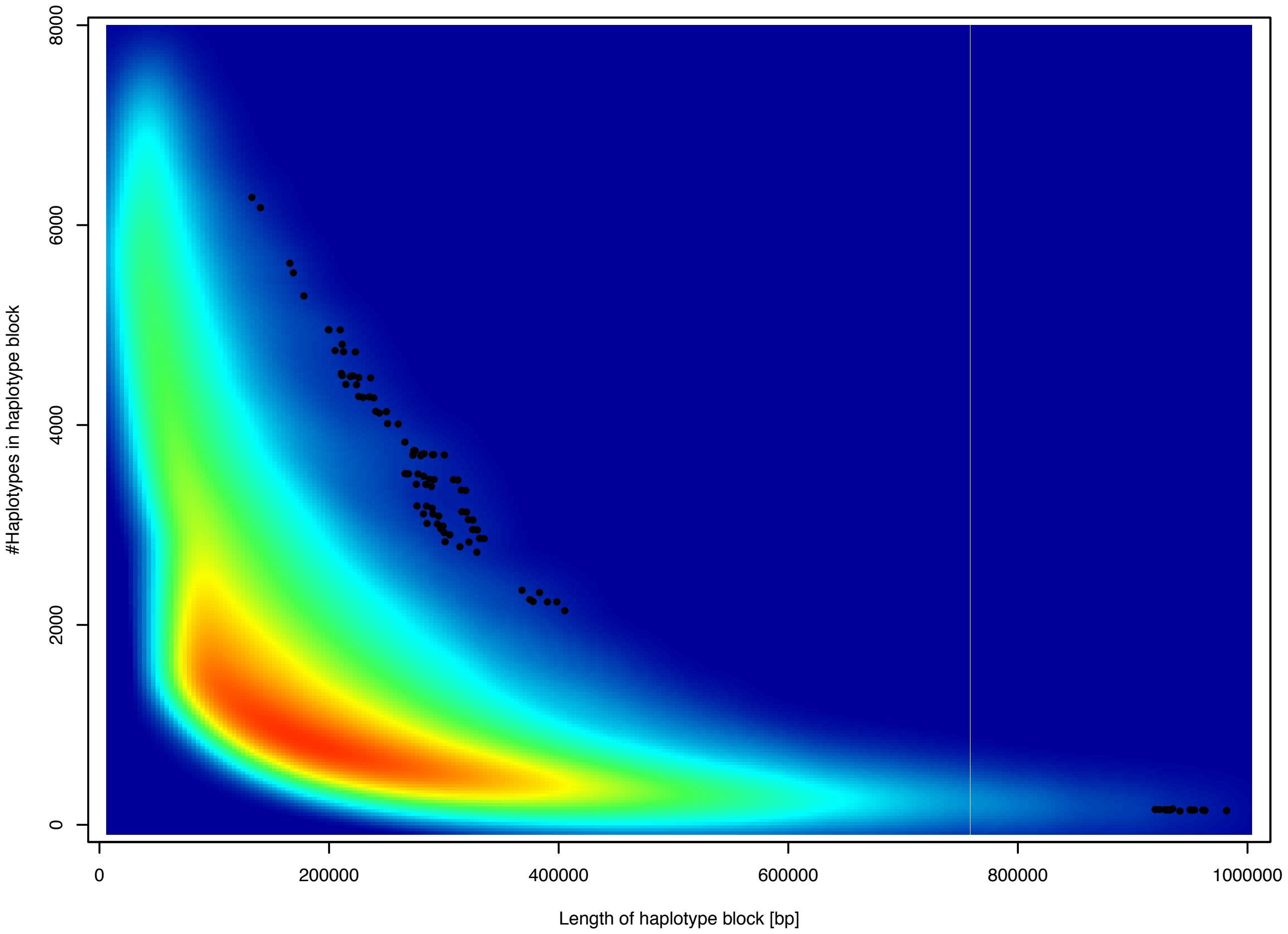

FIG. S21. *Heatmap of joint distribution of haploblock length and number of haplotypes in a HB for HBs found in a neutral simulation of chromosome 2 with bottleneck and exponential population growth downsampled to ~48k SNPs.* See caption of Figure S16 for details.

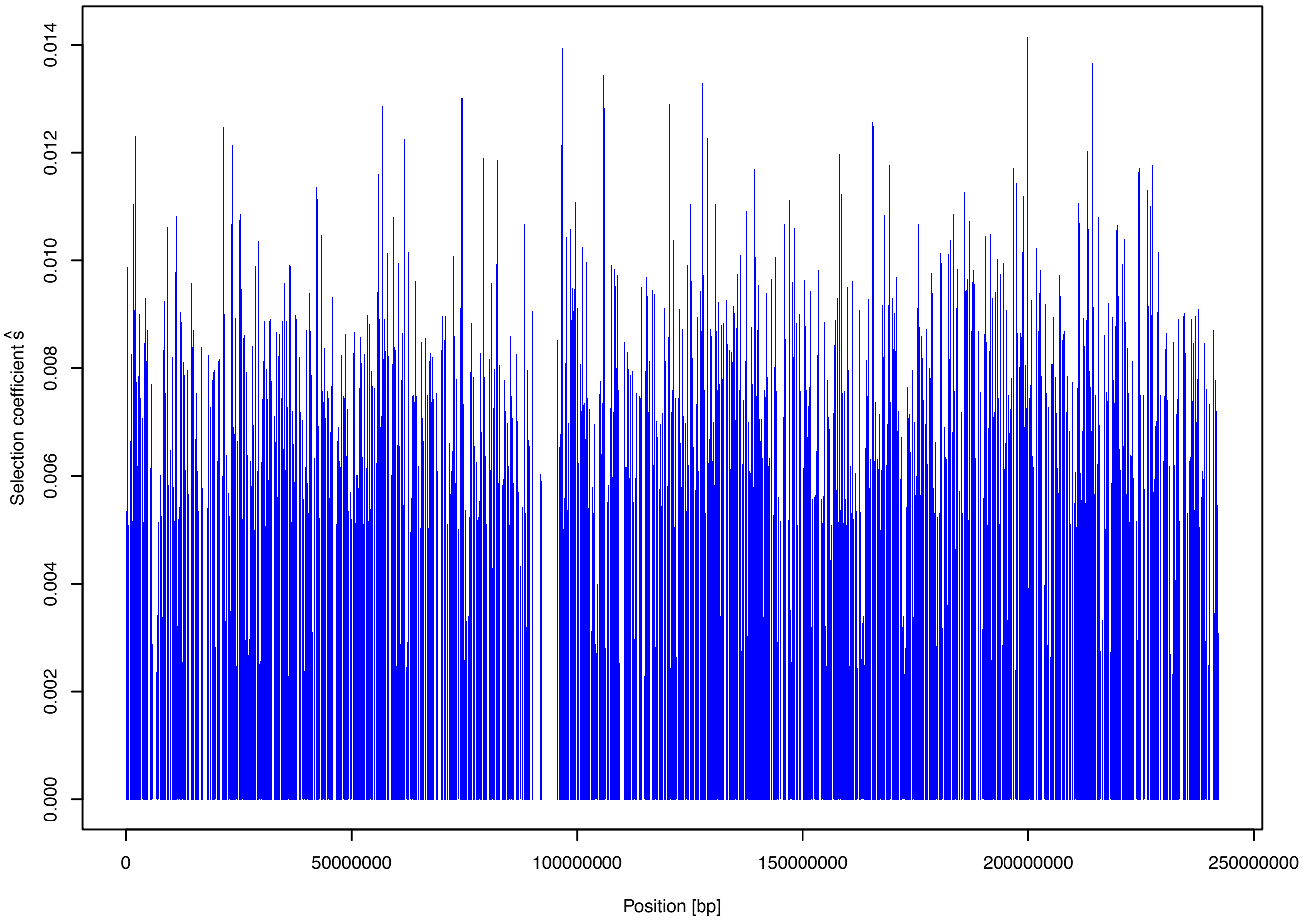

FIG. S22. *Haploblocks found in a neutral simulation of chromosome 2 with constant population size and downsampled to ∼48k SNPs.* The 88,906 blocks that passed both filtering steps are represented analogously to Figure 3.

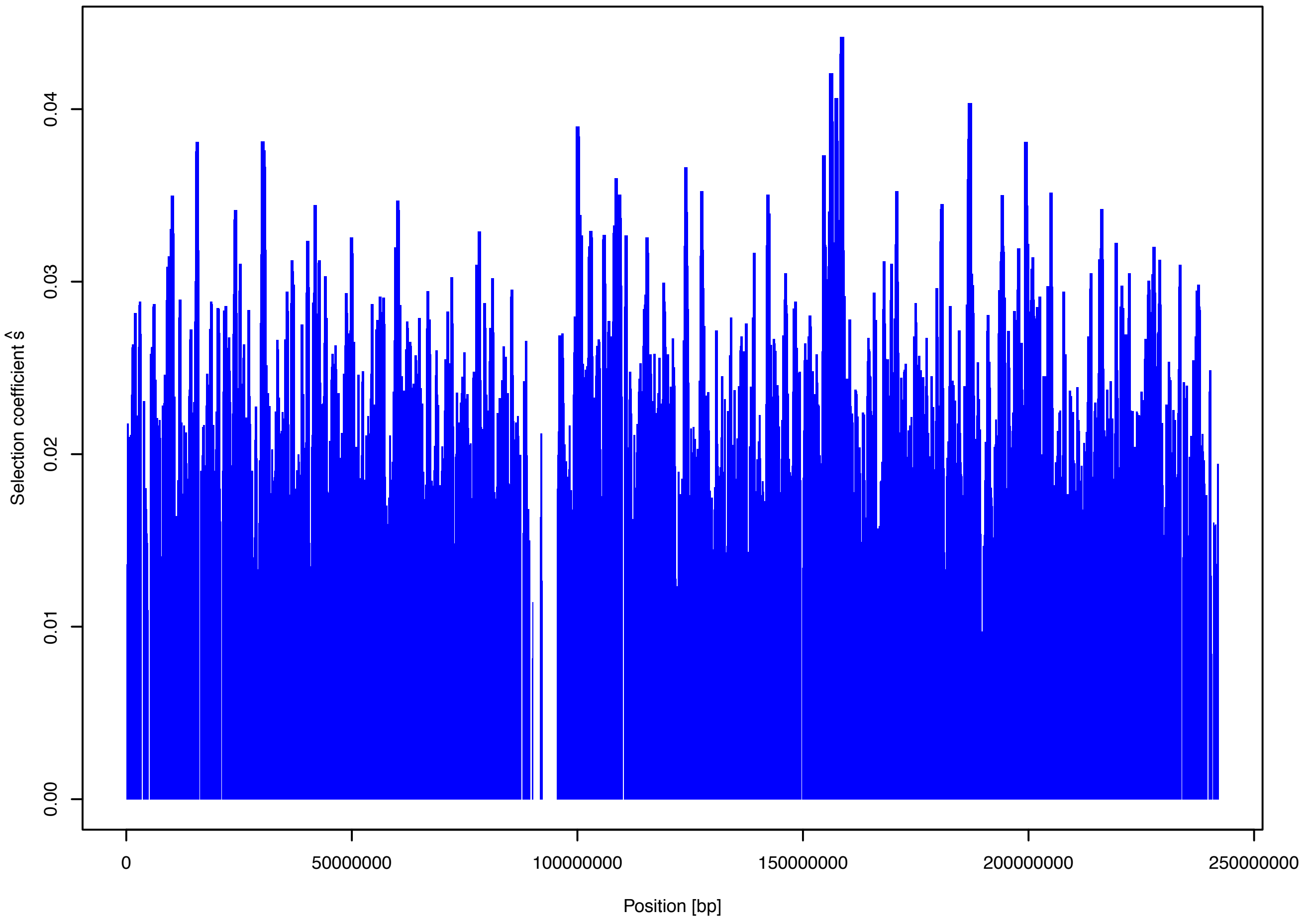

FIG. S23. *Haploblocks found in a neutral simulation of chromosome 2 with bottleneck and exponential population growth downsampled to ∼48k SNPs.* The 1,252,333 blocks that passed both filtering steps are represented analogously to Figure 3.

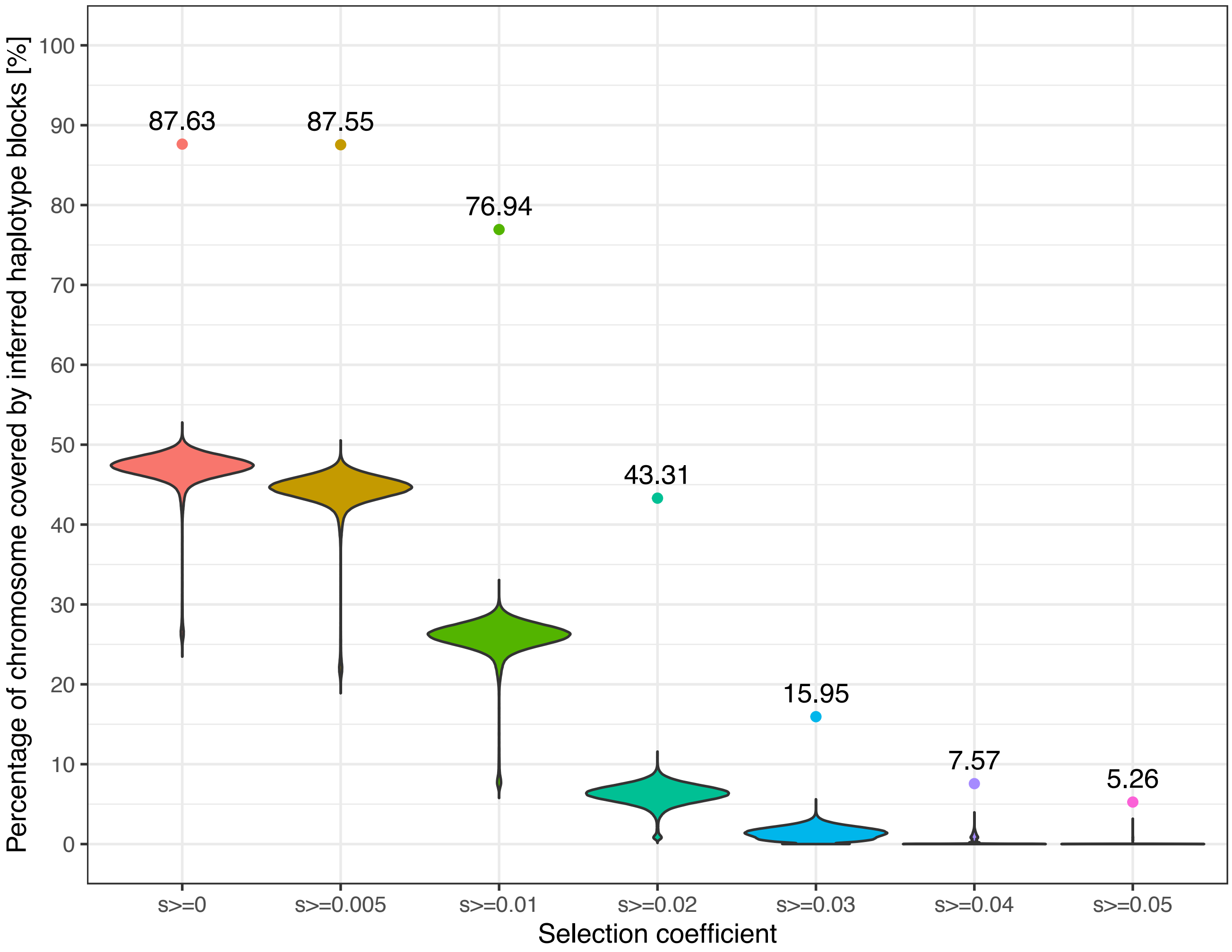

FIG. S24. *Proportion of UK Biobank chromosome 2 covered by selected haplotypes.* Distributions of proportions of individual chromosomes from the UK Biobank dataset covered by haploblocks inferred to be under selection (coverage quantified in base pairs), shown for increasing lower bounds on the selection coefficient. In addition, dots indicate the covered proportion of the chromosome considering all haploblocks found.

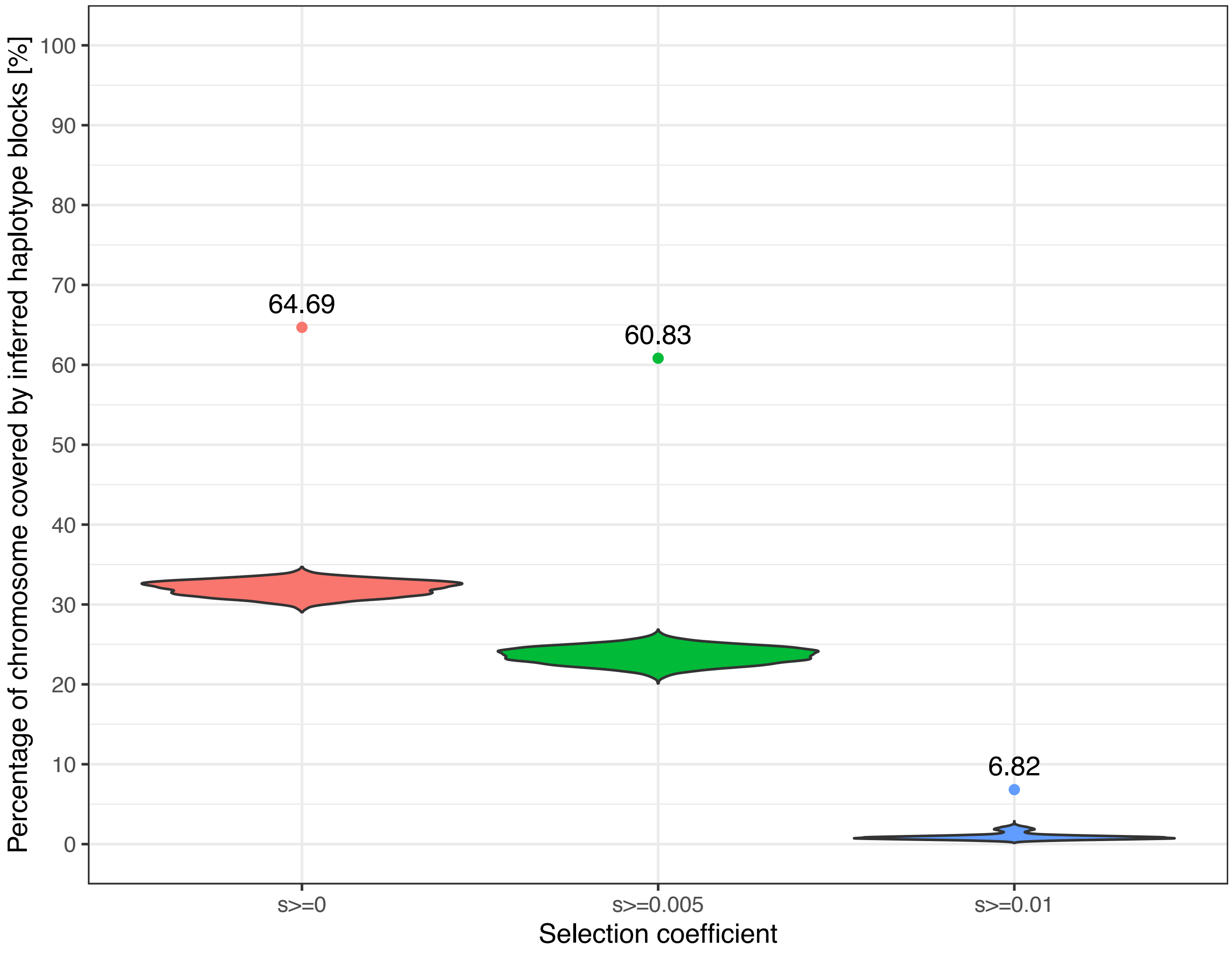

FIG. S25. *Proportion of artificial chromosomes covered by haplotypes inferred to be under selection in a neutral simulation of chromosome 2 with constant population size and downsampled to* $\sim$*48k SNPs.* Results corresponding to the analysis presented in Figure S24 for the 88,906 blocks that passed both filtering steps in the neutral simulation with constant population size mimicking chromosome 2 of the UK Biobank (see Materials and Methods for details on the simulation).

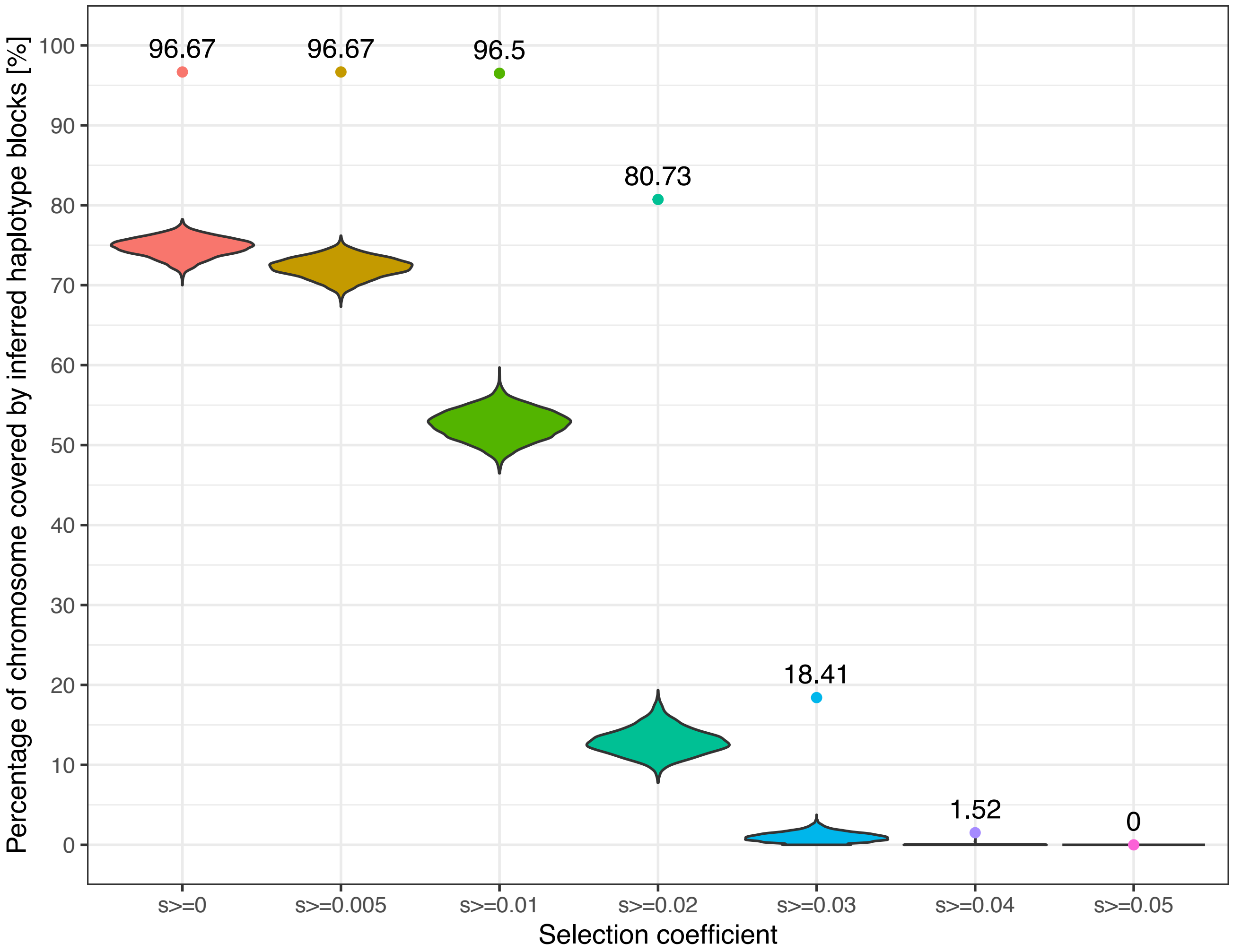

FIG. S26. *Proportion of artificial chromosomes covered by haplotypes inferred to be under selection in a neutral simulation of chromosome 2 with bottleneck and exponential population growth downsampled to ∼48k SNPs.* Results corresponding to the analysis presented in Figure S24 for the 1,252,333 blocks that passed both filtering steps in the neutral simulation with bottleneck and exponential population growth mimicking chromosome 2 of the UK Biobank (see Materials and Methods for details on the simulation).

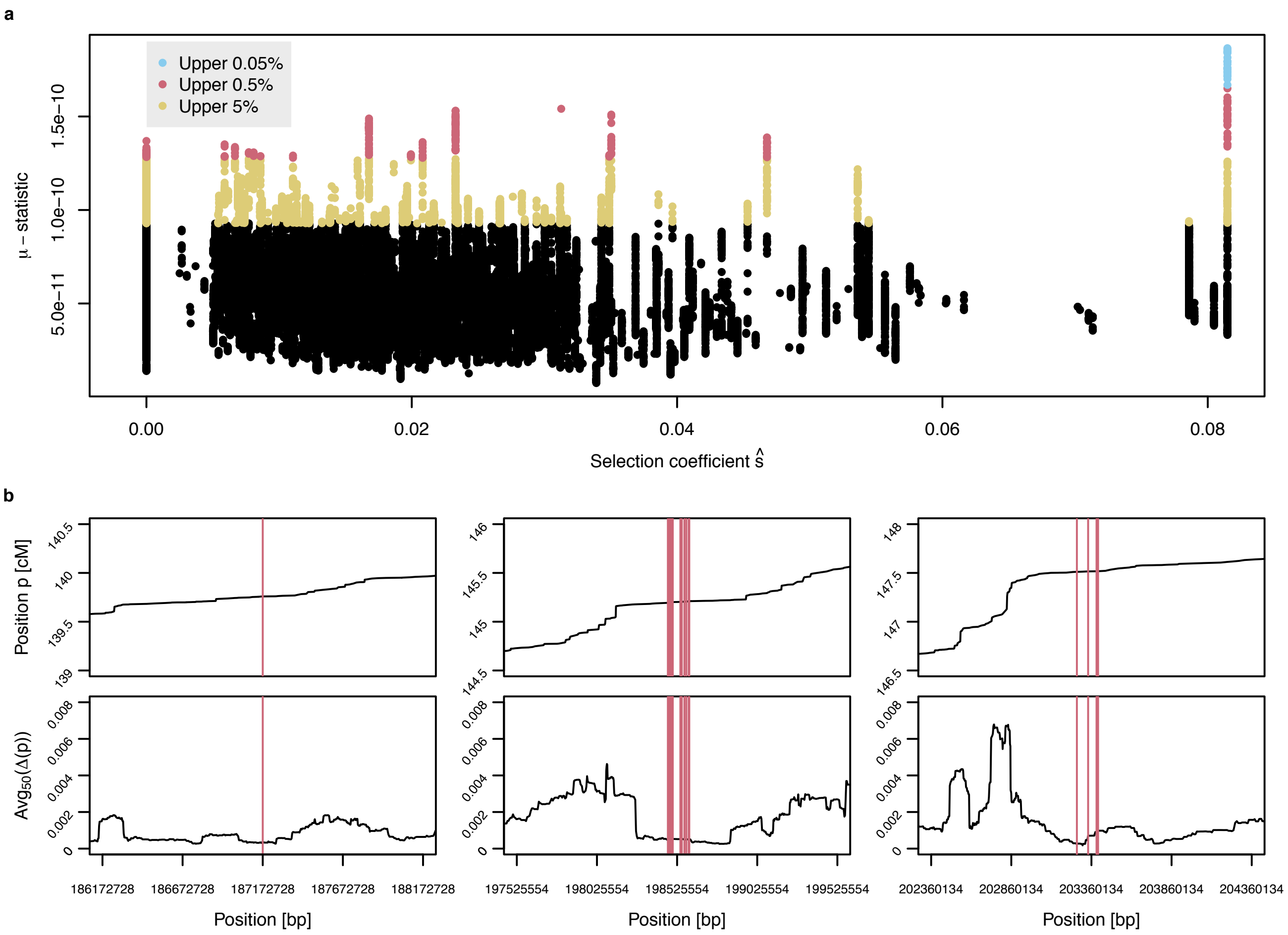

FIG. S27. *Relation between μ-statistic and inferred selection coefficients $\hat{s}$.* (a) shows the relation between μ-statistic and the selection coefficient $\hat{s}$ inferred by HAPLOBLOCKS at corresponding positions. Subfigure (b) shows position on the recombination map (Spence and Song, 2019) (upper panels) and the average difference quotient (lower panels) for the three loci with μ-statistics in the top 0.5% for which HAPLOBLOCKS infers no selection. Difference quotients are averaged over windows of 50 SNPs. Loci flagged by RAiSD are marked by a vertical red line.

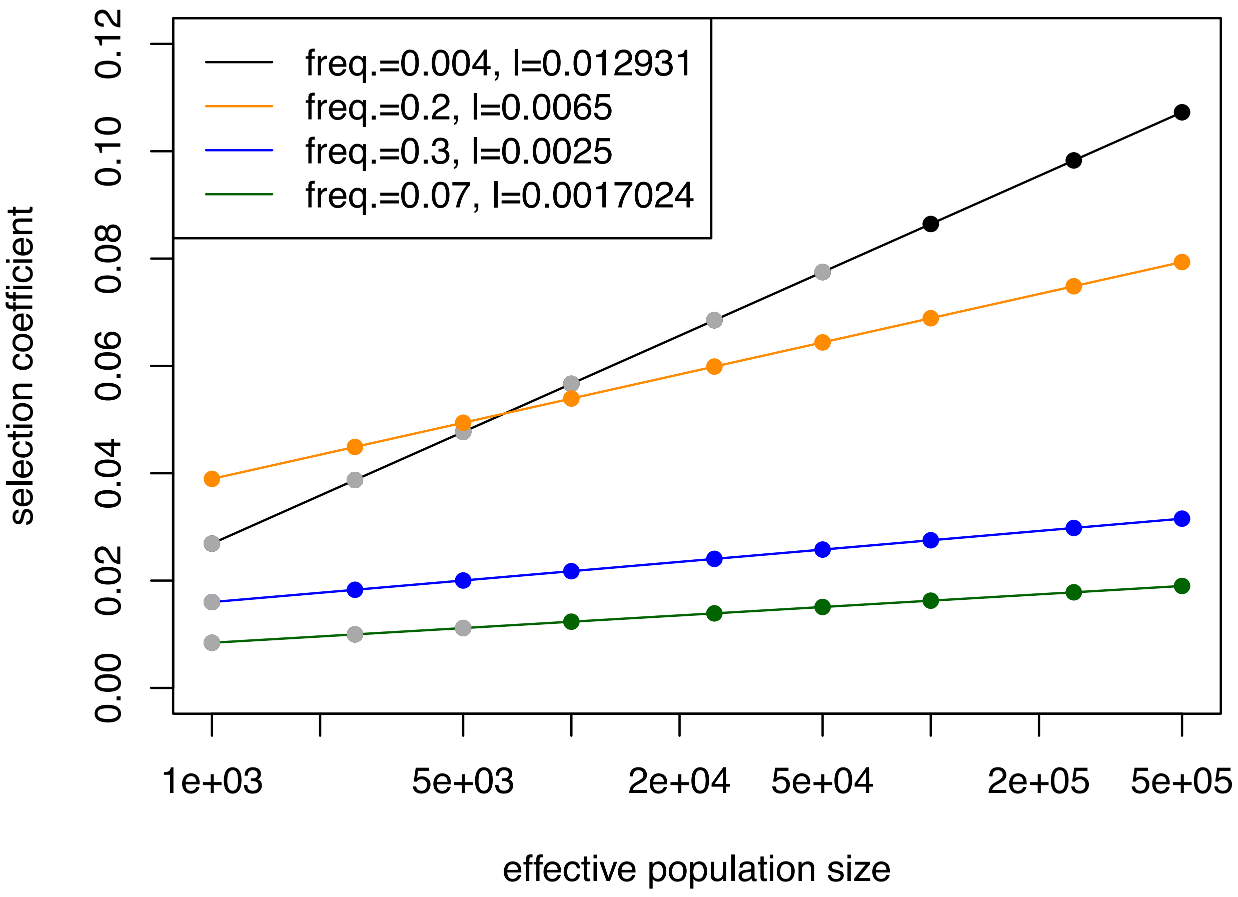

FIG. S28. *Effect of population size parameter.* Assuming $\Delta r = 0.0000013$ cM and 2000 chromosomes overall, we compute $\hat{s}$ (Equation 6) for four hypothetical blocks varying the effective population size parameter. Grey dots indicate that HBs are filtered out. *Abbreviations:* relative frequency (freq.), haploblock length in cM (l.).
